# Identification of CRYAB^+^ KCNN3^+^ SOX9^+^ Astrocyte-Like and EGFR^+^ PDGFRA^+^ OLIG1^+^ Oligodendrocyte-Like Tumoral Cells in Diffuse IDH1-Mutant Gliomas and Implication of NOTCH1 Signalling in Their Genesis

**DOI:** 10.3390/cancers13092107

**Published:** 2021-04-27

**Authors:** Meera Augustus, Donovan Pineau, Franck Aimond, Safa Azar, Davide Lecca, Frédérique Scamps, Sophie Muxel, Amélie Darlix, William Ritchie, Catherine Gozé, Valérie Rigau, Hugues Duffau, Jean-Philippe Hugnot

**Affiliations:** 1 Institut de Génomique Fonctionnelle (IGF), University of Montpellier, Centre National de la Recherche Scientifique (CNRS), Institut National de la Santé et de la Recherche Médicale (INSERM), 34094 Montpellier, France; meera.augustus@inserm.fr (M.A.); donovan.pineau@igf.cnrs.fr (D.P.); Sophie.Muxel@icm.unicancer.fr (S.M.); Amelie.Darlix@icm.unicancer.fr (A.D.); c-goze@chu-montpellier.fr (C.G.); v-rigau@chu-montpellier.fr (V.R.); h-duffau@chu-montpellier.fr (H.D.); 2 Institut des Neurosciences de Montpellier (INM), University of Montpellier, CEDEX 5, 34091 Montpellier, France; franck.aimond@inserm.fr (F.A.); safa.azar@ua.edu.lb (S.A.); frederique.scamps@inserm.fr (F.S.); 3 Department of Pharmaceutical Sciences, Università degli Studi di Milano, 20133 Milan, Italy; davide.lecca@unimi.it; 4 Department of Medical Oncology, Institut Régional du Cancer de Montpellier, University of Montpellier, 34000 Montpellier, France; 5 Institut de Génétique Humaine (IGH), University of Montpellier, CNRS, 34396 Montpellier, France; william.ritchie@igh.cnrs.fr; 6 Laboratory of Solid Tumors Biology, Hôpital Lapeyronie, 371 Avenue du Doyen Giraud, 34295 Montpellier, France; 7 Department of Pathology and Oncobiology, Hôpital Gui de Chauliac, 80 Avenue Augustin Fliche, 34295 Montpellier, France; 8 Neurosurgery Department, Hôpital Gui de Chauliac, 80 Avenue Augustin Fliche, 34295 Montpellier, France; 9 Department of Biology, University of Montpellier, Place Eugène Bataillon, CEDEX 05, 34095 Montpellier, France

**Keywords:** brain tumors, diffuse grade II IDH-mutant glioma, diffuse IDH1-mutant gliomas, cellular heterogeneity, NOTCH1 pathway, BMP

## Abstract

**Simple Summary:**

Diffuse grade II IDH-mutant gliomas are rare brain tumors mainly affecting young patients. These tumors are composed of different populations of tumoral cells. Little is known of these cells and how they are generated. These different cells may show different sensitivity to treatments, so our aim was to study them in detail by directly using patient resections. We identified two clearly distinct tumoral populations and defined reliable markers for them. We also uncovered part of the molecular mechanisms that generate them. Finally, we found that the two cell types have different electrical activity. This article provides unique data and new issues on these rare tumors, which need to be further investigated to develop innovative treatments.

**Abstract:**

Diffuse grade II IDH-mutant gliomas are slow-growing brain tumors that progress into high-grade gliomas. They present intratumoral cell heterogeneity, and no reliable markers are available to distinguish the different cell subtypes. The molecular mechanisms underlying the formation of this cell diversity is also ill-defined. Here, we report that SOX9 and OLIG1 transcription factors, which specifically label astrocytes and oligodendrocytes in the normal brain, revealed the presence of two largely nonoverlapping tumoral populations in IDH1-mutant oligodendrogliomas and astrocytomas. Astrocyte-like SOX9^+^ cells additionally stained for APOE, CRYAB, ID4, KCNN3, while oligodendrocyte-like OLIG1^+^ cells stained for ASCL1, EGFR, IDH1, PDGFRA, PTPRZ1, SOX4, and SOX8. GPR17, an oligodendrocytic marker, was expressed by both cells. These two subpopulations appear to have distinct BMP, NOTCH1, and MAPK active pathways as stainings for BMP4, HEY1, HEY2, p-SMAD1/5 and p-ERK were higher in SOX9^+^ cells. We used primary cultures and a new cell line to explore the influence of NOTCH1 activation and BMP treatment on the IDH1-mutant glioma cell phenotype. This revealed that NOTCH1 globally reduced oligodendrocytic markers and IDH1 expression while upregulating *APOE, CRYAB, HEY1/2,* and an electrophysiologically-active Ca^2+^-activated apamin-sensitive K^+^ channel (KCNN3/SK3). This was accompanied by a reduction in proliferation. Similar effects of NOTCH1 activation were observed in nontumoral human oligodendrocytic cells, which additionally induced strong SOX9 expression. BMP treatment reduced OLIG1/2 expression and strongly upregulated CRYAB and NOGGIN, a negative regulator of BMP. The presence of astrocyte-like SOX9^+^ and oligodendrocyte-like OLIG1^+^ cells in grade II IDH1-mutant gliomas raises new questions about their role in the pathology.

## 1. Introduction

Glioma represents nearly 26% of all central nervous system tumors and 80% of primary malignant brain tumors in adults [1]. Diffuse grade II gliomas [2] are rare tumors accounting for approximately 15% of all gliomas. Their incidence rate is about 1/100,000 person-years. They are slow-growing tumors, primarily occurring in young adults [3,4]. 

Histological diagnosis of these tumors was primarily based on the morphology of tumoral cells (astrocytomas or oligodendrogliomas), but molecular alterations found in these tumors are now incorporated to provide an integrated diagnosis. Indeed, most (>70%) diffuse grade II gliomas carry a recurrent missense mutation in the IDH1 or IDH2 (isocitrate dehydrogenase 1/2) genes, with IDH1R132H being the most commonly identified mutation (90%) [5,6,7,8]. IDH-mutant oligodendrogliomas additionally have an unbalanced translocation of chromosomes 1 and 19, resulting in deletions of 1p and 19q (1p/19q codeletion) [9,10]. Other genetic alterations often found concomitantly in IDH-mutant grade II oligodendrogliomas are *CIC*, *FUBP1*, and *TERT* promoter mutations. IDH-mutant grade II astrocytomas most often carry *ATRX* and *TP53* mutations (abbreviations are listed in Table S5) [11,12].

In addition to genetic abnormalities, there have been reports suggesting the importance of altered signalling pathways in gliomas. This is well documented in high-grade gliomas, with the implication of dysregulated receptor tyrosine kinase signalling (epithelial growth factor (EGFR) [13], platelet-derived growth factor (PDGFR) [14] as well as PI3K-AKT-mTOR and Ras-MAPK pathways [15]. However, there are only a handful of studies exploring the signalling aspects in diffuse grade II IDH-mutant gliomas, referred to here as IDH-DGIIGs [16,17,18,19].

It is now well established that most cancers are comprised of different tumoral cell subtypes, differing, for instance, in their differentiation, proliferating, and metabolic states. IDH-DGIIGs are no exception. Indeed, using immunofluorescence on glioma sections, we previously reported intratumoral heterogeneity in IDH-DGIIGs [19]. This heterogeneity is also supported by two recent single-cell RNA seq studies [20,21]. Additionally, we reported that approximately 20% of IDH-DGIIGs have transformation foci, with cells showing a high level of activation of the STAT3 pathway and reduced levels of a lipid metabolism enzyme, ETNPPL [22]. One consequence of this cellular heterogeneity is that current treatments may only target subpopulations of cells while leaving other cell types unaffected and prone to tumor relapse. It is also not known whether all tumoral cell subtypes have the same ability to proliferate and invade the brain, a major obstacle to treat these tumors. Finally, the intimate molecular mechanisms and pathways underlying the formation of this cellular heterogeneity in IDH-DGIIGs remain ill-defined. 

Clearly, a better description of this cellular heterogeneity and its formation would certainly help define innovative therapeutic strategies. In particular, simple and reliable markers to reveal this cellular heterogeneity in patients would be very useful in routine pathology practices. In this article, we addressed these pending issues by using freshly resected IDH-DGIIG oligodendrogliomas and astrocytomas tumors. Immunofluorescence, primary cell cultures, QPCR, and electrophysiology were used to study cellular heterogeneity and active pathways in these tumors. This revealed the presence of two largely nonoverlapping astrocyte-like and oligodendrocyte-like cell populations, showing different transcriptional factors, active pathways, and receptors. In addition, by conducting functional analysis in vitro, we identified NOTCH1 and BMP pathways as important regulators of the IDH-DGIIG cell phenotype and electrophysiological properties. The presence of two distinct cellular populations in IDH-DGIIGs raises new questions about their role in the pathology, malignant progression, and response to treatments. 

## 2. Materials and Methods

### 2.1. Patient Samples

Cases used in the article are listed in Table S1. Gliomas were classified based on WHO criteria [2] by a neuropathologist (Pr. V Rigau, Hôpital Gui de Chauliac, Montpellier, France). The tumors used in the article are (1) diffuse grade II astrocytomas, showing IDH1 R132H, and TP53 stainings and loss of nuclear staining for ATRX by IHC, and (2) diffuse grade II oligodendrogliomas based on IDH1 R132H IHC staining and 1p19q codeletion. *IDH1* mutation was also confirmed by the sequencing of exon 4, and 1p/19q codeletion was assessed by molecular detection of loss of heterozygosity using polymorphic markers within 1p and 19q chromosome arms, as described previously [23].

### 2.2. Immunofluorescence on IDH-DGIIG Tissues and Cells

**Figure 1:**
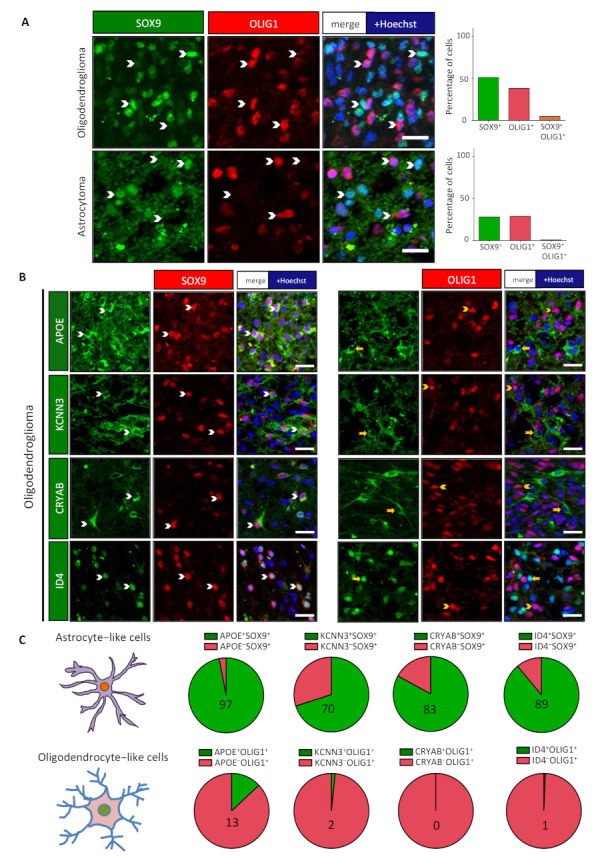
Two nonoverlapping cell subpopulations detected in IDH-DGIIG. (**A**) Immunofluorescence performed on one oligodendroglioma and one astrocytoma using antibodies against SOX9 (green) and OLIG1 (red) revealed the presence of two nonoverlapping cell subpopulations. White arrowheads mark cells expressing either SOX9 or OLIG1 alone. Scale bars = 20 µm. Bar diagrams show the percentage of SOX9^+^ cells (green), OLIG1^+^ cells (red), and percentage of cells double-positive for SOX9^+^ and OLIG1^+^ (orange) among the total number of cells. The two subpopulations were also detected in other cases (see Figure S2A). (**B**) SOX9^+^ cells show specific protein expression. Double stainings for APOE, CRYAB, KCNN3, and ID4 with SOX9 or OLIG1 showed their preferential expression in SOX9^+^ cells. White arrowheads identify double-positive cells, while yellow arrowheads/arrows represent single positive cells. Scale bars = 20 µm. (**C**) Pie diagrams representing the percentage of double-positive (green) and single-positive (red) cells in SOX9^+^ (upper lane) and OLIG1^+^ (lower lane) populations. Numbers indicate the percentage of double-positive cells.

Freshly resected tumors were fixed using 4% paraformaldehyde for 1 h at room temperature (RT), followed by cryopreservation using successive sucrose solutions (10%, 20%, 30%). Tumors were cryosectioned (10 µm), and sections were washed with 0.1 M PBS–glycine to reduce background signals. Permeabilization and blocking were performed with 0.3% Triton X-100 and 10% donkey serum (Sigma, St. Quentin Fallavier, France), followed by overnight incubation with primary antibodies (see Table S4 for the list of antibodies and dilutions) and a one-hour incubation with Alexa 488− or Cy3-conjugated secondary antibodies (Jackson ImmunoResearch, Pennsylvania, US). No primary antibody or antibody against GFP was used as negative control. Nuclei were counterstained using Hoechst 33342. A minimum of 300 cells was counted to distinguish the two nonoverlapping SOX9^+^ and OLIG1^+^ subpopulations (Figure 1A and Figure S2A). A minimum of 100 cells was counted to determine the coexpression of specific markers in SOX9^+^ and OLIG1^+^ cells. Countings were made in tumor areas containing high cellular density while avoiding infiltrative areas, where the proportion of nontumoral cells can be significant. For cell cultures, coverslips were fixed using 4% paraformaldehyde for 20 min, and immunofluorescences were performed, as for tumor sections, with 0.1% Triton X-100. For staining with O4 antibody, the permeabilization step was omitted. Images were taken using a Zeiss Apotome Z2 microscope.

### 2.3. Cell Cultures Derived from IDH-DGIIG Resections

Dissociation of resected tumors was done for 30 min at 37 °C using trypsin (Sigma #T4799, 13 mg/mL), hyaluronidase (Sigma, #H3884, 7 mg/mL) and DNase I (Roche, Sigma, St. Quentin Fallavier, France, #10104159001, 10 mg/mL), then stopped by a trypsin inhibitor (Sigma, #T9003, 50 mg/mL). Cells were passed through a 70 µm cell strainer (Miltenyi Biotech, Paris, France) and purified by a two-step Percoll (Sigma, #GE17-0891-02) density gradient to remove myelin and red blood cells. Microglia cells were removed, and O4^+^ cells were collected by sorting with CD11b^+^ and O4^+^ magnetic microbeads (Miltenyi Biotech), respectively, according to the manufacturer’s protocol. O4^+^ cells were grown on culture vessels coated with poly-D-lysine (Sigma, #P7886; 25 µg/mL) and laminin (Sigma, #L2020, 2 µg/cm^2^). Cells were cultured in DMEM/F12 1:1 media (Gibco, Fisher scientific, Illkirch CEDEX, France) supplemented with N2 (Thermo Fisher, Fisher scientific, Illkirch CEDEX, France #17502048), D-glucose (Sigma, 0.6%), L-glutamine (Thermo Fisher, #25030024, 2 mM), B27 w/o vitamin A (Invitrogen, Fisher scientific, Illkirch CEDEX, France), EGF (Peprotech, Neuilly-sur-Seine, France, 20 ng/mL), FGF2 (Peprotech, 10 ng/mL), PDGFA (Peprotech, 20 ng/mL), and heparin (Sigma, 2 µg/mL). The LGG275 cell line was isolated by serial passaging of an IDH-DGIIG culture showing proliferative cells, which was derived from an ATRX/IDH1 R132H mutated resection (obtained from the Montpellier hospital biological resource bank, with authorization from the Montpellier hospital Institutional Review Board (IRB ID: 198711; N° IRB-MTP_2019_IRB_MTP_10_15). 

### 2.4. Lentivirus Infection, RNA Extraction, and QPCR

O4^+^-purified cells and LGG275 cells were infected with either a control-YFP or NICD-YFP virus (gift from Dr. Sutton, Yale, New Haven, CT, USA; multiplicity of infection = 6). NICD cDNA codes for amino acids from 1762 to 2556 of human NOTCH1 [24]. Five days after transduction, RNAs were extracted using the Arcturus Picopure RNA kit (Thermo Fisher), and 100–200 ng of cDNA was synthesized with random primers and reverse transcriptase (Promega, Charbonnières-les-Bains, France, GoScript). Quantitative PCR was performed using 2.5 ng of cDNA in duplicates. Primers (Sigma) are listed in Table S3. The KAPA SYBR PCR kit (Sigma) was used for QPCR (Light Cycler 480, Roche). Gene expression was calculated using the 2^−ΔΔCt^ method, using β-actin (ACTB) for normalization. For inhibitor experiments, LGG275 cells were treated with γ-secretase inhibitors (DAPT (GSI-IX, Selleck Chemicals, Breda, Netherlands) and LY411575 (Peprotech)) at 10 µM for 5 days before RNAs were extracted for QPCR. For the DLL4 ligand experiment (Figure S13A), plates were coated with DLL4 (5 µg/mL; Peprotech) or BSA at 4 °C overnight before seeding the cells. RNAs were extracted after 4 days. For BMP signalling experiments described in Figure 6C, cells were treated with 10 ng/mL of BMP2 or BMP4 (Peprotech) for 5 days with the addition of new cytokines every 2 days. 

### 2.5. Electrophysiology

KCNN3/SK3 channel currents were measured in LGG275 cells transduced with control-YFP or NICD-YFP viruses 3 days after the infection. For electrophysiology, cells were placed in bathing (extracellular) solution composed of 147 mM NaCl, 5 mM KCl, 2 mM CaCl2, 1.5 mM MgCl2, 10 mM HEPES, 10 mM glucose, pH 7.4. Recording pipettes filled with a solution containing 135 mM K-methane-sulfonate, 0.1 mM EGTA, 8 mM KCl, 10 mM HEPES, 2 mM MgATP, 0.5 mM NaGTP, pH 7.3 were sealed to the cell membrane. KCNN3/SK3 current was recorded by applying a 600 ms electrical ramp from −80 mV holding potential, gradually increasing up to +60 mV in both control-YFP and NICD-YFP transduced cells. Ionomycin-induced and apamin-sensitive KCNN3/SK3 current densities were measured by adding 10 µM ionomycin and 1 µM apamin to the bathing solution, respectively. All recordings were performed at RT using an Axopatch 200B amplifier and a Digidata 1322A A/D board (Molecular Devices, Berkshire, UK) and acquired at 5 kHz. Current analysis was performed using Clampfit version 10 (Axon Instruments, Molecular Devices).

### 2.6. Western Blot Analysis

Tissue samples were lysed using RIPA lysis buffer (Sigma), containing 5 mM NaF, 0.5 mM Na-vanadate, and 1X-protease inhibitor cocktail (Roche). Samples were incubated on ice for 30 min and centrifuged for 20 min at 13,000 rpm at 4 °C; the protein concentration in the supernatant was measured with a DC protein assay (BioRad, Grabels, France). Samples were separated by SDS-PAGE and transferred on a 0.2 µm PVDF membrane (BioRad). After blocking with 5% nonfat dried milk in TBST (Tris-buffered saline, 0.1% Tween 20), primary antibodies listed in Table S4 were incubated overnight at 4 °C and revealed using peroxidase-conjugated antibodies and an ECL kit (BioRad). Images were captured using the ChemiDocTM XRS Imaging system (Biorad). 

### 2.7. Statistical Analysis

All experiments were performed at least three times for confirmation. Data are represented as mean ± standard error of mean (SEM). Statistical differences in experiments were analyzed with the tests indicated in the legends using GraphPad 6 Prism software. Unpaired two-tailed *t*-tests with equal standard deviation were used for QPCR analysis. *, **, ***, **** represent *p* < 0.05, <0.01, <0.001 and <0.0001 significance, respectively. 

## 3. Results

### 3.1. Two Distinct Populations of Astrocyte-Like and Oligodendrocyte-Like Tumoral Cells in IDH-DGIIG 

In order to distinguish several cell populations in IDH-DGIIG tumors, we examined three diffuse grade II astrocytoma tumors with IDH1 R132H and ATRX mutations and three diffuse grade II oligodendroglioma tumors with an IDH1 R132H mutation and a 1p19q codeletion (Table S1). To identify two potential cell types in these samples, we used OLIG1 and SOX9 as well-known markers for oligodendrocytic and astrocytic-lineage cells, respectively (Figure S1A,B). Figure 1A and Figure S2A show that in the six examined tumors, two largely nonoverlapping populations of OLIG1^+^ and SOX9^+^ cells were present. To ascertain that these two cell populations were indeed tumoral, we performed costainings for SOX9 and OLIG1 with (i) ATRX for one astrocytoma and (ii) a specific antibody for the mutated form of IDH1 (IDH1 R132H) in one oligodendroglioma. Figure S2B shows that in the studied astrocytoma, no nuclear staining for ATRX was observed in the majority (>90%) of OLIG1^+^ and SOX9^+^ cells, which are, thus, tumoral (Figure S2B,C). For the oligodendroglioma, >90% of the OLIG1^+^ cells were costained with IDH1 R132H, whereas only around 15% of the SOX9^+^ cells showed a faint IDH1 R132H staining (Figure S2B,C). However, as indicated below, this number is probably an underestimation, as the level of expression of the IDH1 enzyme appears lower in SOX9^+^ cells than in OLIG1^+^ cells.

To ascertain that SOX9^+^ cells show some astrocytic features, further stainings were performed. We selected proteins that are expressed in diffuse low-grade gliomas, for which literature and database analyses (Figure S1A,B and Table S2) show a clear preferential expression in the astrocytic lineage. We chose APOE, ID4, KCNN3 astrocytic proteins, for which reliable antibodies were used in combination with OLIG1 and SOX9 transcription factors. We also analyzed the expression of CRYAB (alpha-B crystallin) as this protein was described to be elevated up to 22-fold in IDH1-mutant gliomas [25]. Stainings for these four proteins were performed in one oligodendroglioma and one astrocytoma mutated for IDH1, along with 1p/19q codeletion or ATRX mutation, respecttively. Results presented in Figure 1B,C (oligodendroglioma) and Figure S3 (astrocytoma) show that the expression of these four proteins is more associated with SOX9^+^ cells than OLIG1^+^ cells. 

**Figure 2:**
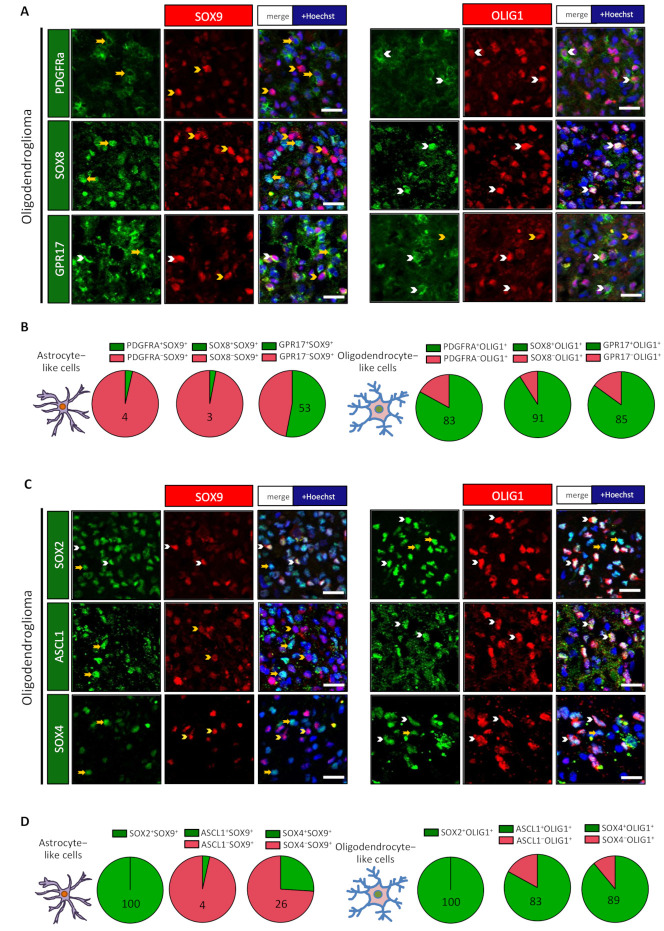
OLIG1^+^ cells express proteins associated with oligodendrocyte lineage and neural precursor cells. Double immunofluorescences for indicated proteins on one oligodendroglioma. White arrowheads identify double-positive cells, while yellow arrowheads/arrows represent single-positive cells. Scale bars = 20 µm. (**A**) Double stainings for GPR17, PDGFRα, and SOX8 with SOX9 or OLIG1 revealed their preferential association with OLIG1^+^ cells in one oligodendroglioma. (**B**,**D**) Pie diagrams representing the percentage of double-positive (green) and single-positive (red) cells in SOX9^+^ and OLIG1^+^ populations. Numbers indicate the percentage of double-positive cells. (**C**) Double stainings for ASCL1, SOX2, and SOX4 with SOX9 or OLIG1 revealed the preferential association of ASCL1 and SOX4 with OLIG1^+^ cells in one oligodendroglioma, while SOX2 was expressed by both populations.

We then performed the same analysis with proteins that are hallmarks of oligodendrocytic lineage, namely, GPR17, PDGFRA, and SOX8 (Figure S1A,B and Table S2). PDGFRA and SOX8 were clearly more associated with OLIG1^+^ cells in both the oligodendroglioma and the astrocytoma (Figure 2A,B and Figure S4A,B). For GPR17, its expression was more confined to OLIG1^+^ cells in the oligodendroglioma (Figure 2A,B), whereas a similar expression was found in the SOX9^+^ and OLIG1^+^ subpopulations in the explored astrocytoma (Figure S4A,B). 

Collectively, these results indicate the presence of at least two cell populations in IDH-DGIIG, showing an astrocyte-like and oligodendrocyte-like phenotype. 

### 3.2. Astrocyte-Like and Oligodendrocyte-Like Cells Have Different Levels of Receptors and Signalling Proteins

Considering the distinct phenotype of SOX9^+^ and OLIG1^+^ cells, we reasoned that these cells might present different active pathways, receptors, and neural developmental transcription factors. We started addressing this issue by performing costainings for SOX9 and OLIG1 with other transcription factors that are highly expressed by neural precursor cells during brain development: ASCL1 (also known as MASH1), SOX2, and SOX4. We found that SOX2 was expressed at the same level in both cell types (Figure 2C,D and Figure S4C,D). On the contrary, ASCL1 and SOX4 expression were clearly more expressed in OLIG1^+^ cells in both the explored oligodendroglioma (Figure 2C,D) and astrocytoma (Figure S4C,D) samples. 

**Figure 3:**
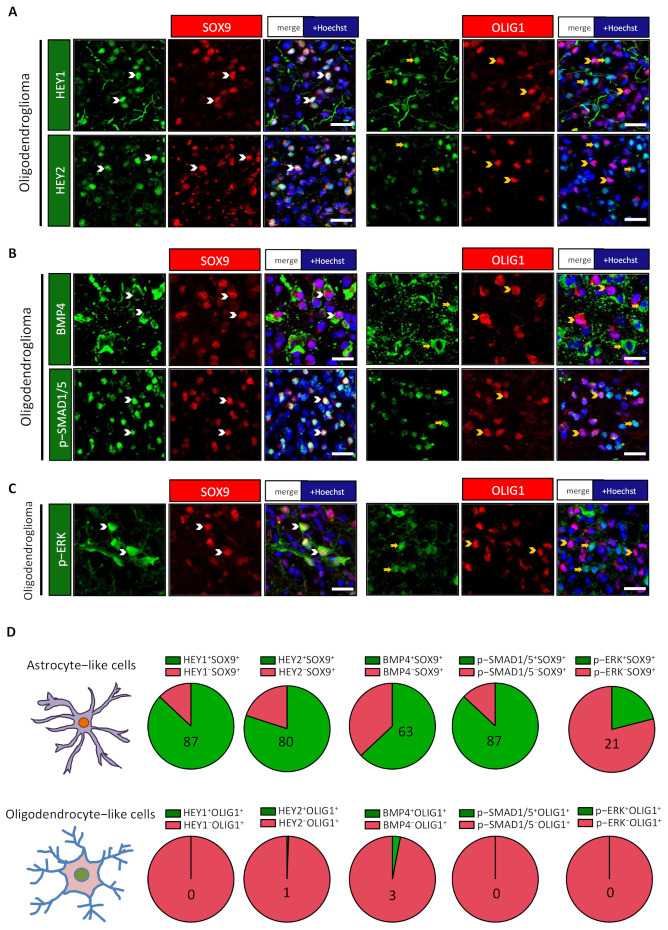
SOX9^+^ cells express specific signalling proteins and transcription factors. (**A**–**C**) Double immunofluorescences in one oligodendroglioma for HEY1, HEY2, BMP4, p-SMAD1/5, and p-ERK with SOX9 and OLIG1 revealed their preferential expression in SOX9^+^ cells. White arrowheads identify double-positive cells, while yellow arrowheads/arrows show single-positive cells. Scale bars = 20 µm. (**D**) Pie diagrams representing the percentage of double-positive (green) and single-positive (red) cells in SOX9^+^ and OLIG1^+^ cells. Numbers indicate the percentage of double-positive cells.

We next studied the expression of proteins that are considered as good readouts of activation of BMP, NOTCH1, and ERK/MAPK pathways. We previously reported that in glioblastomas, NOTCH1 pathway activation led to the upregulation of HEY1 and HEY2 transcription factors [26]. This observation led us to assess HEY1 and HEY2 stainings in the two cellular populations. The results, presented in Figure 3A,D for one oligodendroglioma and Figure S5A,D for one astrocytoma, indicate that HEY1 and HEY2 are preferentially expressed in SOX9^+^ cells, suggesting that NOTCH1 signalling could be active in these cells. With regard to BMP signalling, this pathway is often associated with the generation of astrocytes during brain development and in several pathological situations [27,28]. We looked for activation of this pathway by performing costainings for SOX9 and OLIG1 with the phosphorylated form of SMAD1/5 (p-SMAD1/5), a signalling protein downstream of the BMP pathway. We found that p-SMAD1/5 was preferentially detected in the SOX9^+^ population (Figure 3B,D and Figure S5B,D). This suggested that glioma cells might express BMP RNA and protein. Indeed, the mining of two databases indicated significant upregulation of BMP2 in grade II/III oligodendrogliomas and astrocytomas compared to nontumoral tissues; BMP4 was overexpressed in oligodendrogliomas in one database (Figure S6A). Correlation analysis also showed that BMP2 and BMP4 expression are positively correlated in these tumors (Figure S6B). We confirmed this result in our samples by QPCR and WB for BMP2 and 4. The results presented in Figure S7A,B show significant upregulation of both BMP2 and BMP4 mRNA in the five explored samples and their strong correlated expression. BMP2 and BMP4 proteins were detected by Western blot analysis in all examined grade II/III tumors (Figure S7C). To study which cell types express BMP2 and 4 in IDH-DGIIG, we performed IF for these proteins. We could not obtain a reliable staining for BMP2 using different antibodies; however, we detected a preferential expression of BMP4 in astrocyte-like SOX9^+^ cells (Figure 3B,D for one oligodendroglioma and Figure S5B,D for one astrocytoma). 

**Figure 4:**
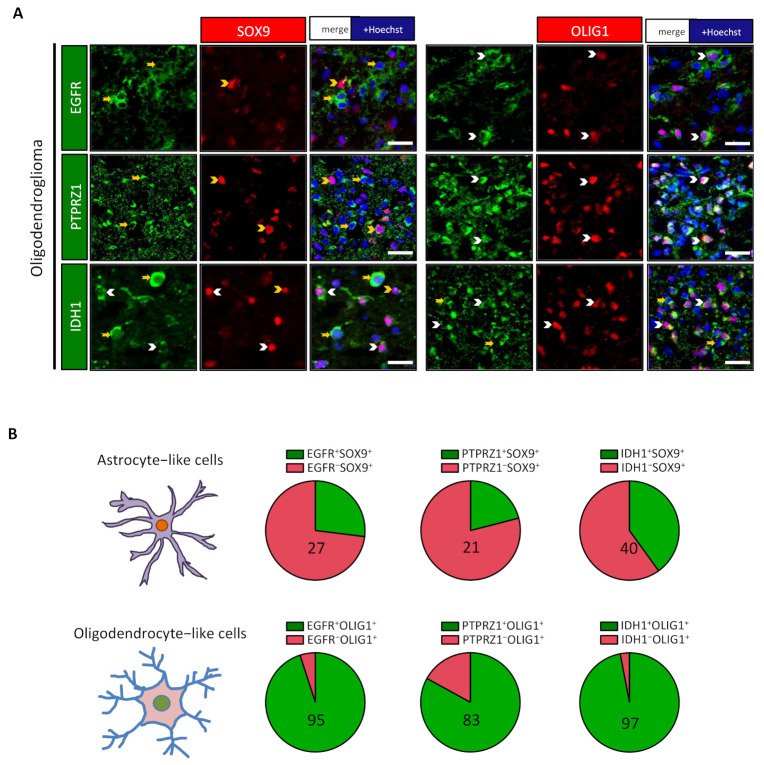
OLIG1^+^ cells express specific receptors and IDH1. (**A**) Double immunofluorescences in one oligodendroglioma for EGFR, PTPRZ1, and IDH1 with SOX9 and OLIG1 revealed their preferential expression with OLIG1^+^ cells. White arrowheads identify double-positive cells, while yellow arrowheads/arrows represent single-positive cells. Scale bars = 20 µm. (**B**) Pie diagrams representing the percentage of double-positive (green) and single-positive (red) cells in SOX9^+^ and OLIG1^+^ cells. Numbers indicate the percentage of double-positive cells.

The receptors for EGF (EGFR) and PTN (PTPRZ1) have important roles in the genesis of gliomas [18,29,30,31,32,33,34]. We thus examined their expression in the two cell populations. Surprisingly, we found that these two receptors were more expressed in OLIG1^+^ cells in the explored oligodendroglioma (Figure 4A,B) and astrocytoma (Figure S8A,B). Activation of EGFR and other receptors activates the ERK/MAPK pathway, leading to phosphorylation of the ERK protein (p-ERK). We thus questioned the presence of p-ERK in the two populations by IF. The results presented in Figure 3C,D and Figure S5C,D show that the SOX9^+^ population appears to have higher activation of the ERK/MAPK pathway. 

Altogether, these results show that the two identified cell populations in IDH-DGIIG express different levels of receptors and signalling pathways. 

### 3.3. Astrocyte-Like and Oligodendrocyte-Like Cells Express Different Levels of IDH1 Enzyme 

IDH1 is a cytoplasmic enzyme that converts isocitrate into alpha-ketoglutarate [35]. Unexpectedly, an exploration of mouse and human brain single-cell databases showed that the IDH1 gene is preferentially expressed by oligodendrocytic lineage cells (Figure S9). This observation prompted us to explore the presence of the IDH1 enzyme in SOX9^+^ and OLIG1^+^ cells by IF, with an antibody raised against the wild-type enzyme. Figure 4A,B shows that in the explored oligodendroglioma, IDH1 staining was preferentially associated with the OLIG1^+^ cell population. In contrast, in the studied astrocytoma, the expression of IDH1 was similarly detected in both OLIG1^+^ and SOX9^+^ populations (Figure S8A,B).

### 3.4. NOTCH1 Activation Modifies IDH-DGIIG Cells’ Phenotype and Reduces Their Proliferation

The identification of at least two clearly distinct cell types in IDH-DGIIG questions how these cells are generated. We hypothesized that the NOTCH1 and BMP pathways may influence the IDH-DGIIG cell phenotype, especially as we found that SOX9^+^ and OLIG1^+^ cells have a differential expression of proteins typically involved in these signallings (HEY1/2 and p-SMAD1/5). To evaluate this possibility, we derived primary cultures from IDH-DGIIG resections mutated for ATRX and IDH1 R132H (Figure S10). These cultures were established by magnetic sorting for the O4^+^ antigen that is expressed by both immature oligodendrocytes and bipotent astro-oligodendrocyte progenitors in the brain [36]. The presence of tumoral cells in the cultures was evaluated by IF against IDH1 R132H and ATRX. Resected samples can contain both tumoral and nontumoral territories in variable proportion, especially in supratotal resections [37], and, consequently, we found that the percentage of IDH1 R132H^+^ ATRX^−^ cells was highly variable between cultures, ranging from 5% to 90%. When few mutated cells were present, the cultures mainly consisted of highly-branched cells (Figure S11A,B) with small nuclei (perimeter = 22.9 µm ± 0.3, *n* = 200 cells), expressing a high level of ATRX, CNP, OLIG1, O4, and SOX10, whereas stainings for IDH1 R132H, EGFR, GFAP, and SOX9 were rare or absent in these cells (Figure S11C,D). Considering their morphology and their markers, these cells are very likely to be nontumoral oligodendrocytic cells. In contrast, in cultures containing lots of tumoral cells (Figure S10A,B), we noted that these have a larger and often abnormal nucleus (perimeter = 30.7 µm ± 0.4, *n* = 200 cells) and express high levels of EGFR, OLIG1, and SOX9 and weak stainings for CNP and SOX10 (Figure S10C,D). Compared to the in vivo situation, we could not identify two clearly distinct SOX9^+^ and OLIG1^+^ tumoral cells in these cultures, and the majority of cells coexpressed OLIG1 and SOX9 (Figure S10C, Lane 4). This could be due to phenotypic modifications induced by cell culture conditions, as observed, for instance, in cultured neural stem cells [38]. 

**Figure 5:**
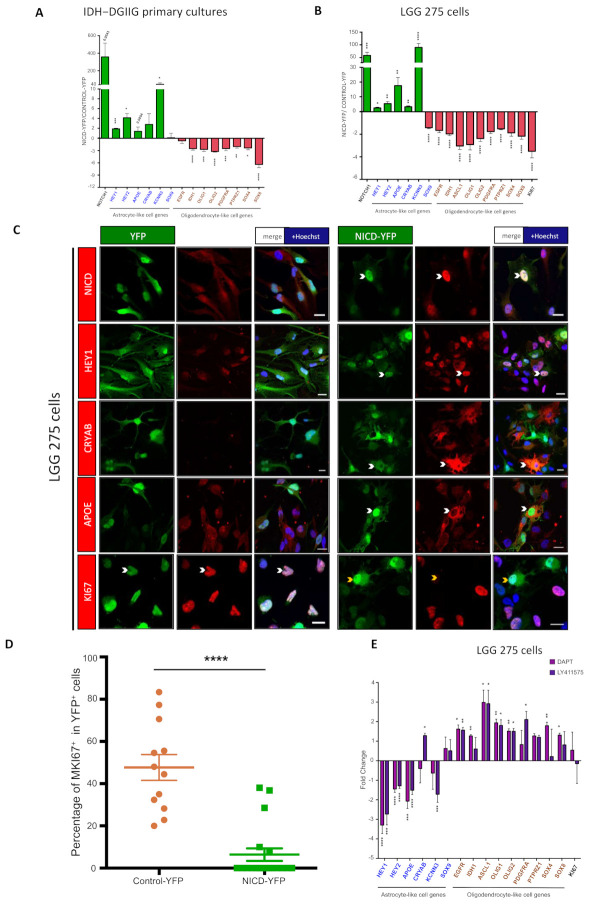
NOTCH1 activation modifies IDH-DGIIG cell phenotype and reduces proliferation. (**A**,**B**) QPCR analysis for indicated genes in O4-purified primary cultures containing a high % of tumoral cells (>70%; *n* = 4 cases, astrocytomas) (**A**) and in LGG275 cells (*n* = 5 independent experiments) (**B**). Values represent the mean ± SEM of gene expression fold change observed in cells transduced with NICD-YFP vs. YFP lentiviruses. Genes in blue and brown are markers found preferentially associated with SOX9^+^ and OLIG1^+^ cells, respectively, on IDH-DGIIG sections. Tests = two-tailed *t*-tests. (**C**) Immunofluorescence for indicated proteins in YFP or NICD-YFP transduced LGG275 cells. White and yellow arrowheads show YFP^+^ cells that are positive or negative for the assessed protein, respectively. Note the nuclear localization of the NOTCH1 activated form (NICD) after transduction with NICD-YFP lentivirus. Scale bars = 20 µm. (**D**) Quantification of MKI67^+^ cells in LGG275 cells transduced with YFP and NICD-YFP lentiviruses. Values represent the mean ± SEM of the percentage of MKI67^+^ cells observed in YFP^+^ cells (*n* = 12 fields, 2 independent experiments). Test = two-tailed *t*-test. (**E**) QPCR analysis for indicated genes in LGG275 cells treated with NOTCH1 signalling inhibitors (DAPT, LY411575, 10 µM) for 5 days (*n* = 4 independent experiments). Values represent the mean ± SEM of gene expression fold change observed in treated vs. control cells. Tests = two-tailed *t*-tests. Genes in blue and brown are markers found preferentially associated with SOX9^+^ and OLIG1^+^ cells, respectively, on IDH-DGIIG sections. Tests = two-tailed *t*-tests. *, **, ***, **** represent *p* <0.05, <0.01, <0.001 and <0.0001 significance respectively.

To test the influence of the NOTCH1 pathway on IDH-DGIIG cells, we selected four independent cultures containing >70% of IDH1 R132H ATRX mutated cells. Cells were infected with a lentivirus expressing the constitutively active form of NOTCH1 (Notch intracellular domain, NICD) and YFP, which resulted in strong upregulation of NOTCH1 mRNA expression (Figure 5A). We analyzed by QPCR the expression of most of the genes that we found differentially expressed in SOX9^+^ and OLIG1^+^ cells on the IDH-DGIIG sections. These are *EGFR, IDH1, OLIG1, PDGFRA, PTPRZ1,* and *SOX4/8* for OLIG1^+^ cells and *APOE, CRYAB, HEY1/2, KCNN3,* and *SOX9* for SOX9^+^ cells. The *OLIG2* gene was also included in the analysis as this transcription factor is often coexpressed with *OLIG1* in oligodendrocytic cells [39]. The results presented in Figure 5A indicate that 7/8 of the genes associated with OLIG1^+^ cells were significantly reduced by NICD expression (*IDH1, OLIG1/2, PDGFRA, PTPRZ1, SOX4/8*), while 3/6 of the genes preferentially expressed in SOX9^+^ cells (*HEY1/2, KCNN3*) were significantly upregulated. No significant influence of NICD on SOX9 was detected, which was, in fact, already highly expressed in these cultures (Figure S10C, lane 4). *APOE* showed upregulation (fold change range = 1.3–2.6) in three of four explored primary cultures but did not reach significance. 

**Figure 6:**
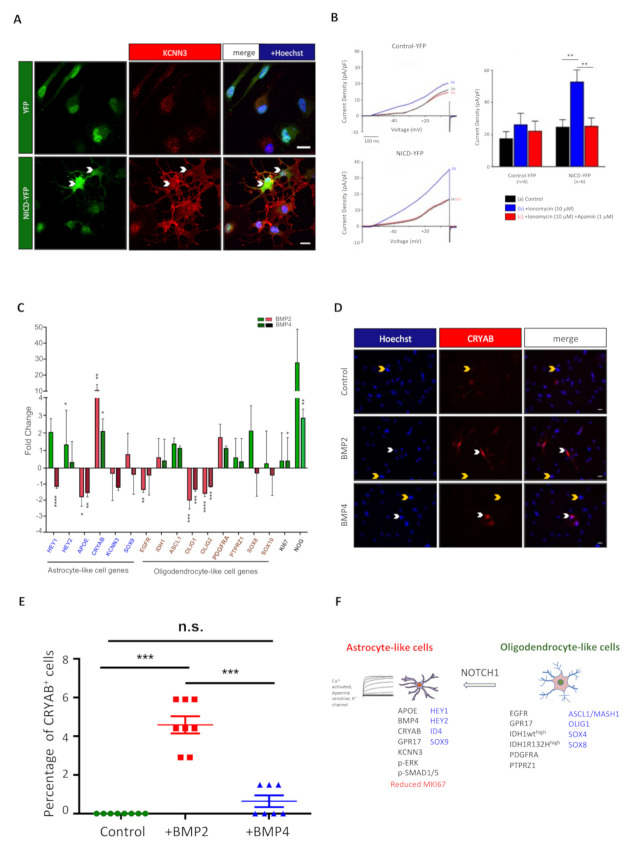
NOTCH1 induces KCCN3/SK3 channels. BMP influence on IDH-DGIIG cell phenotype. (**A**) Immunofluorescence for KCNN3/SK3 channels in YFP and NICD-YFP transduced LGG275 cells. Changes in cellular morphology upon NOTCH1 activation are also visible. Scale bars = 20 µm. (**B**) KCNN3/SK3 currents induced by NOTCH1 signalling. Channel activity was studied in YFP^+^ cells using the whole-cell patch-clamp technique under a voltage-clamp configuration. Left panels: Representative currents recorded by applying a 600 ms electrical ramp from −80 mV holding potential to +50 mV in control-YFP (top) and NICD-YFP transduced (bottom) cells in the three indicated conditions (a,b,c). An increase in current density was observed in the presence of 10 µM ionomycin (to increase the intracellular Ca2^+^ level) in YFP-NICD transduced cells (blue curve). This ionomycin-induced current increase was specifically blocked by 1 µM apamin (a specific SK channel blocker; red curve). Right panels: Bar charts showing the means ± SEM of current densities obtained at +40 mV from current/voltage curves obtained after 500 ms voltage steps from −80 mV holding potential to +40 mV every 10 mV in YFP and YFP-NICD transduced cells. Ionomycin-induced and apamin-sensitive current densities were significantly higher in YFP-NICD transduced cells compared to control cells (*n* = 6 cells, 3 independent experiments, test = two-tailed *t*-test). (**C**) QPCR analysis for indicated genes in LGG275 cells treated for BMP2 or BMP4 (10 ng/mL) for 5 days (*n* = 4 independent experiments). Values represent the mean ± SEM of gene expression fold change observed in treated vs. control cells. Genes in blue and brown are markers found preferentially associated with SOX9^+^ and OLIG1^+^ cells, respectively, on IDH-DGIIG sections. Tests = two-tailed *t*-tests. (**D**) Immunofluorescence for CRYAB in control or BMP2/4-treated LGG275 cells. White and yellow arrowheads show CRYAB^+^ and CRYAB^−^ cells, respectively. Scale bars = 10 µm. (**E**) Quantification of CRYAB^+^ cells after BMP2/4 treatment. Values represent the mean ± SEM of a percentage of CRYAB^+^ cells in control or treated cells. (*n* = 7 fields, 3 coverslips). (**F**) Graphical summary of main results. Transcription factors are in blue. *, **, ***, **** represent *p* <0.05, <0.01, <0.001 and <0.0001 significance respectively.

To confirm these results, we used a cell line (named LGG275) that was derived from a diffuse low-grade glioma patient with *IDH1* and *ATRX* mutations (Table S1). This cell line contains bipolar and multipolar cells (Figure S12A) expressing IDH1 R132H but not ATRX (Figure S12B, Lanes 1,3) that grow very slowly (doubling time = 9.6 ± 0.1 days, *n* = 6 wells). Figure S12B,C shows that LGG275 cells express both SOX9 and OLIG1 proteins (Lane 4) together with EGFR, CNP, and SOX10 (Lanes 1–3), as seen in primary tumoral cultures (Figure S10). These cells were transduced with YFP and YFP-NICD lentiviruses, which led to strong upregulation of *NOTCH1* RNA (Figure 5B) and a clear nuclear localization of the NICD fragment (Figure 5C). NICD in these cells downregulated 9/9 of oligodendrocytic genes (*ASCL1, EGFR, IDH1, OLIG1/2, PDGFRA, PTPRZ1,* and *SOX4/8*) and upregulated 5/6 of astrocytic genes (*APOE, CRYAB, HEY1/2, KCNN3*). *SOX9* was moderately but significantly downregulated by NICD in this cell line. We could confirm the upregulation of APOE, CRYAB, HEY1, and KCNN3 proteins by NICD by IF (Figure 5C and Figure 6A). In order to explore the effect of NOTCH1 activation in a more physiological setup, we exposed LGG275 cells to DLL4, a NOTCH1 ligand predominantly expressed in endothelial cells [40,41]. This resulted in the significant upregulation of *APOE, HEY1,* and *KCNN3,* while the expression of all oligodendrocytic genes was reduced (Figure S13A). The influence of NOTCH1 signalling was also monitored using two γ-secretase inhibitors (DAPT and LY411575) that block NOTCH1 receptor cleavage, thereby preventing signal activation. Figure 5E shows that these inhibitors downregulate *APOE, HEY1/2*, and *KCNN3* expression while globally increasing oligodendrocytic genes (*ASCL1, EGFR, IDH1, OLIG1/2, PDGFRA, SOX4/8*).

Finally, as we previously found that NOTCH1 activation drastically reduced the rate of proliferation of glioblastoma stem cells [26], we also assessed the effect of NICD on the proliferation of LGG275 cells by measuring the expression of the proliferation marker MKI67 by IF and QPCR. As presented in Figure 5B–D, we observed that NICD expression led to a sharp reduction in proliferation. 

Collectively, these results show a major role for NOTCH1 signalling in controlling the phenotype and proliferation of IDH-DGIIG cells. 

### 3.5. NOTCH1 Activation Modifies Electrophysiological Properties of IDH-DGIIG Cells 

Among the astrocytic genes upregulated by NOTCH1 activation, the strongest effect was observed for *KCNN3,* with a fold change of 30 and 100 in primary IDH-DGIIG cells and the LGG275 line, respectively. KCNN3 (also known as SK3) is a small conductance K^+^ channel activated by Ca2^+^, which can be specifically blocked by the bee venom apamin [42]. This channel is expressed in the brain, but its role in astrocytes and gliomas is currently unknown. To see whether NICD could elicit KCNN3/SK3 currents, we performed electrophysiology on control-YFP or NICD-transduced LGG275 cells. The results presented in Figure 6B show that in NICD-transduced cells, an increase in Ca2^+^ by ionomycin leads to an elevated outward K^+^ current, which is blocked by apamin. This indicates that NICD can induce a functional expression of KCNN3 channels in IDH-DGIIG cells. 

### 3.6. NOTCH1 Activation Induces SOX9 in Human Oligodendrocytic Cells

We were surprised not to observe an upregulation of *SOX9* by NOTCH1 activation in IDH-DGIIG tumoral primary cultures and LGG275 cells (Figure 5A,B) as this gene is upregulated by NOTCH1 in other contexts [43]. We reasoned that, in fact, the high level and, maybe, already saturated expression of *SOX9* in these cells (Figures S10C and S12B) might preclude an upregulation after NICD transduction. To test this hypothesis, we used three primary cultures derived from IDH-DGIIG supratotal resections, which contained mostly (>90%) nontumoral SOX10^high^ oligodendrocytic cells (Figure S11). In these cultures, SOX9 could be barely detected by IF (Figure S11C; Lane 6). QPCR for *SOX9* validated this low level of expression compared to tumoral IDH-DGIIG primary cultures and LGG275 cells (Figure S13B). Figure S13E shows that overexpression of NICD in these cells induced a sharp upregulation of *SOX9* (fold change = 14.8) that could also be observed at the protein level by IF (Figure S13C,D). As observed in LGG275 and tumoral IDH-DGIIG primary cultures, NICD overexpression in these cells also reduced oligodendrocytic gene expression while increasing *CRYAB, HEY1/2,* and *KCNN3* (Figure S13E). 

### 3.7. BMPs Influence on IDH-DGIIG Cell Phenotype

BMP proteins are important regulators of neural precursor fate by controlling their phenotype and proliferation [27]. BMP can also induce astrocytic differentiation of oligodendroglioma propagating cells [28]. Their effect not only hinges on the canonical Smad pathway but also involves the NOTCH1 pathway and HEY1/2 transcription factors [44]. The high expression of BMP proteins found in IDH-DGIIG tumors, especially in astrocyte-like cells (Figure 3B,D and Figure S5B,D), prompted us to determine the effect of BMP2/4 on the LGG275 cell phenotype. The cells were treated with BMP2 or 4 for 5 days, and their phenotype was assessed by QPCR as done for NOTCH1. The results in Figure 6C show that the effects were much less contrasted than for NOTCH1, and only *OLIG1/2* and *APOE* were significantly downregulated by both cytokines. One remarkable variation was observed for *CRYAB*, which was upregulated 10− and 2-fold by BMP2 and BMP4 treatments, respectively (Figure 6C). Correspondingly, the CRYAB protein, which was not detected in control LGG275 cells by IF, became clearly present in a fraction of cells exposed to BMP2 and, to a lesser extent, BMP4 (Figure 6D,E). Last, mining of the diffuse low-grade glioma subset of the TCGA database shows that expression of BMP2/4 is highly correlated to the expression of the BMP-inhibitor NOG (NOGGIN) gene (Figure S6A,B). We confirmed this tight correlation by performing QPCR for NOG in the five diffuse low-grade glioma resections we previously analyzed for BMP2/4 (Figure S7A,B). This suggested that BMP2 or 4 cytokines might control NOG expression in IDH-DGIIG. Indeed, using QPCR, we found that BMP2 was a powerful regulator of NOG in LGG275 cells, with a 30-fold increase (Figure 6C). BMP4 could also significantly upregulate NOG, but only by 3-fold (Figure 6C).

## 4. Discussion

In this article, we studied tumoral cell heterogeneity in diffuse grade II IDH-mutant gliomas, for which little is known at the cellular and pathway levels. We used primary cell cultures, a new cell line, and sections directly derived from freshly resected samples to maximize relevance to the pathology. We provide evidence for the existence of two main nonoverlapping populations in these tumors that can be easily distinguished on the basis of differential expression of OLIG1 and SOX9. We used double-stainings to further characterize these cells and found that SOX9^+^ cells are related to the astrocytic lineage while OLIG1^+^ cells are more associated with the oligodendrocyte lineage. The phenotype of these two cell types is summarized in Figure 6F and differs in several aspects. At the transcriptional level, astrocyte-like cells express high levels of HEY1/2, ID4, and SOX9, while oligodendrocyte-like cells display strong stainings for ASCL1, OLIG1, and SOX4/SOX8. We also identified that oligodendrocyte-like cells had higher levels of three receptors (EGFR, PDGFRA, and PTPRZ1) than astrocyte-like cells. Finally, we observed a higher level of APOE, BMP4, KCNN3/SK3, and CRYAB proteins in astrocyte-like cells, whereas the IDH1 enzyme appears to be more expressed in oligodendrocyte-like cells in oligodendroglioma. SOX9^+^ and OLIG1^+^ cells were observed in 6/6 of the samples we explored, suggesting that they are likely to be present in a majority of IDH-DGIIG. These two cell types account for over 50% of tumoral cells, but their relative proportion is variable between cases (Figure 1A and Figure S2A).

Former studies based on electron microscopy [45] and immunohistochemistry of diffuse low-grade gliomas [46,47,48] have described the presence of phenotypic heterogeneity within tumoral cells. In particular, in oligodendrogliomas, subpopulations of gliofibrillary oligodendrocytes have been reported [49,50,51]. While this work was performed, the cellular composition of IDH-DGIIG was also explored using the recent technological approach based on single-cell RNA seq [20,21]. These studies reported the presence of three subpopulations of malignant cells: nonproliferating cells differentiated along the astrocytic and oligodendrocytic lineages and proliferative undifferentiated cells that resemble neural stem/progenitor cells. Collectively, the present immunofluorescence analysis, former electron microscopic/IHC studies, and single-cell RNA sequencing approaches all converge to identify the presence of astrocyte-like and oligodendrocyte-like cells in IDH-DGIIG. In the normal brain, oligodendrocytes and astrocytes have very distinct properties. For instance, they differ at the metabolic, angiogenic, and migration levels. It remains to be established whether oligodendrocyte-like and astrocyte-like cells found in IDH-DGIIG show similar dichotomic properties. 

One unexpected finding is the highest expression of IDH1 enzyme in oligodendrocyte-like cells than in astrocyte-like cells in oligodendroglioma. This was observed using an antibody for the mutated form of IDH1 (R132H) but also confirmed with an antibody against wild-type IDH1. In fact, in accordance with these findings, single-cell RNA databases for human and mouse adult brain cells revealed that IDH1 is more expressed in the oligodendrocytic lineage, especially in immature oligodendrocytes, than in the astrocytic lineage (Figure S9). We also found that IDH1 expression is also negatively regulated by the activation of NOTCH1 signalling (Figure 5A,B), which represses several oligodendrocytic genes in IDH-DGIIG cells. What could be the function of IDH1 in OLIG1^+^ tumoral cells? Compared to IDH2, which is located in the mitochondria to generate alpha-ketoglutarate and NADH, H^+^ in the Krebs cycle, the IDH1 enzyme is located in the cytoplasm, where it generates alpha-ketoglutarate and NADPH, H^+^. NADPH, H^+^ is a key metabolite for lipid and myelin synthesis [52], and, thus, the high expression of IDH1 observed in the oligodendrocyte lineage may serve the requirement for high lipid metabolism in these cells.

Another unexpected finding is the specific expression of CRYAB in astrocyte-like tumoral cells. This crystallin protein is typically expressed in inflammatory contexts such as multiple sclerosis [53] and has antiapoptotic, neuroprotective, and anti-inflammatory functions [54]. In cancer, CRYAB can act as an oncoprotein or a tumor suppressor [54]. *CRYAB* is a transcriptional target of BMP signalling [55], and accordingly, we found it to be strongly upregulated by BMP signalling (Figure 6C–E). *CRYAB* is also upregulated by NICD overexpression in LGG275 cells (Figure 5B), suggesting a potential double regulation by NOTCH1 and BMP signalling. A potential link between CRYAB, NOTCH1, and BMP pathways is also supported by diffuse low-grade glioma RNA profiles in the TCGA database, showing a significant correlation between *CRYAB, BMP2*, and *HEY2* expression (Figure S14). The role of CRYAB in IDH-DGIIG cells is not known and remains to be elucidated. 

The discovery of astrocyte-like and oligodendrocyte-like tumoral cells prompted us to analyze the expression of the G protein-coupled receptor GPR17. In normal human and mouse brains, GPR17 is almost exclusively expressed by oligodendrocyte progenitors and committed oligodendrocytic cells [56]. It participates in the differentiation and maturation process of oligodendrocytes. Considering its expression profile, we expected GPR17 to be only expressed in OLIG1^+^ cells; however, we found it to be also present in a substantial number of SOX9^+^ astrocyte-like cells. In an inflammatory context, GPR17 can be expressed in nonoligodendrocytic cells [56], so a similar phenomenon could occur in IDH-DGIIG. Considering that GPR17 activation can impede progenitor differentiation [56], its expression in astrocyte-like and oligodendrocyte-like cells may contribute to the differentiation blockage observed in IDH-DGIIG cells [57]. 

Using IDH-DGIIG primary cultures and a new *IDH1/ATRX* mutated cell line, we found that the NOTCH1 pathway deeply influences the IDH-DGIIG cell phenotype. Indeed, by manipulating this pathway through different approaches (constitutively activated form, pharmacology, and DLL4 ligand), we found that NOTCH1 activation globally reduced the expression of oligodendrocytic genes while concomitantly increasing *KCNN3* and *APOE*, which are typically associated with the astrocytic lineage. This was accompanied by a reduction of proliferation in LGG275 cells, as previously seen in high-grade gliomas [26,58]. Using nontumoral human O4-purified oligodendrocytic cells, we also found that the activation of NOTCH1 reduced oligodendrocytic gene expression while upregulating *CRYAB, KCNN3,* and *SOX9*. These results are consistent with the literature showing that NOTCH1 activation in neural precursor cells promotes astroglial lineage entry [59] and blocks oligodendrocyte differentiation [60]. Considering the high expression of SOX9 in IDH-DGIIG astrocyte-like cells (Figure 1A and Figure S2A) and that SOX9 is a target of NOTCH1 during nervous system development [43,61] and in other non-neural tissues [62,63], we also expected *SOX9* expression to be upregulated by NOTCH1 activation in IDH-DGIIG primary cultures and the LGG275 cell line. This was, however, not observed in these cells, which, in fact, already expressed a high and maybe saturated level of *SOX9* (Figures S10C and S12B). This may result from EGF receptor activation by EGF in the media, which can upregulate SOX9, as seen in other cell types [64]. In contrast, in nontumoral oligodendrocytic O4^+^ cells, which do not express SOX9 (Figure S11C), both SOX9 gene and protein were strongly induced by NOTCH1 activation. To our knowledge, this is the first demonstration of a NOTCH1-induced SOX9 expression in human oligodendrocytic cells. Besides modifying their phenotype, we also found that NOTCH1 activation modifies the electrophysiological properties of IDH-DGIIG cells as NICD overexpression induces the expression of electrophysiologically-active KCNN3/SK3 channels. Considering the high fold change (between 30 to 100) in KCNN3 expression induced by NOTCH1 activation, it is possible that this channel is a direct target of this pathway. Ca2^+^-activated K^+^ channels are involved in membrane hyperpolarization but also regulate cellular shape, migration, and proliferation [65]. Likewise, they may also regulate these properties in IDH-DGIIG cells. Lastly, we found that NOTCH1 activation reduces cell proliferation in the cell line we isolated. This result fuels the emerging notion that the Notch pathway acts as a tumor suppressor in IDH-DGIIG [58]. 

Despite the presence of a high expression of BMP proteins in IDH-DGIIG (Figure S7C), especially in astrocyte-like cells for BMP4 (Figure 3B and Figure S5B), the effect of BMP2/4 treatment on the genes we explored was less prominent than for NOTCH1 activation. Only the expression of CRYAB was strongly upregulated. We also found strong induction of *NOGGIN* by BMP proteins, probably reflecting the presence of a negative retro control for this pathway in IDH-DGIIG cells. With regards to CRYAB, BMPs protect endothelial cells from apoptosis, in part, by upregulating CRYAB, which acts as an antiapoptotic factor [55]; a similar situation could occur in IDH-DGIIG cells. 

## 5. Conclusions

In conclusion, the identification of astrocyte-like SOX9^+^ and oligodendrocyte-like OLIG1^+^ tumoral cells in IDH-DGIIG raises several new questions. Do these cells show the same sensitivity to treatment? Do they have the same ability to invade the brain, a major obstacle to treating these tumors? Can they interconvert in the tumor? These issues merit further investigation to derive new therapeutic strategies against these tumors.

## Data Availability

The data presented in this study are available in this article and Supplementary Materials.

## Supplementary Materials

The following are available online at https://www.mdpi.com/article/10.3390/cancers13092107/s1, Figure S1: Preferential astrocytic and oligodendrocytic expression of markers selected in the study; Figure S2: Two nonoverlapping cell subpopulations detected in IDH-DGIIG; Figure S3: SOX9+ cells show specific protein expression; Figure S4: OLIG1+ cells express proteins associated with the oligodendrocytic lineage and neural precursor markers; Figure S5: SOX9+ cells specifically express various signalling molecules and receptors; Figure S6: Expression profiles for BMP2/4 and NOGGIN mRNA in diffuse low-grade glioma databases; Figure S7: Expression of BMP2/4 and NOGGIN in diffuse low-grade gliomas; Figure S8: OLIG1+ cells specifically express/activate various receptors and IDH1; Figure S9: Cell-type-specific expression of IDH1 in adult mouse and human brains; Figure S10: Phenotypic characterization of tumoral O4+ cells isolated from IDH-DGIIG resections; Figure S11: Phenotypic characterization of nontumoral O4+ cells isolated from IDH-DGIIG samples; Figure S12: Phenotypic characterization of the LGG275 cell line. Figure S13: DLL4 effect on cell phenotype and regulation of SOX9 by NOTCH1 signalling; Figure S14: Expression correlation of CRYAB, HEY2, and BMP2 in the TCGA database. Table S1: Detailed information of the cases used in the article; Table S2: List of cellular markers and supporting references for their specific expression; Table S3: PCR primer pairs used for quantitative RT–PCR; Table S4: Antibodies used for immunostaining; Table S5: List of abbreviations used in the article.
