# Supplementary material for: Identification of CRYAB+ KCNN3+ SOX9+ Astrocyte-Like and EGFR+ PDGFRA+ OLIG1+ Oligodendrocyte-Like Tumoral Cells in Diffuse IDH1-Mutant Gliomas and Implication of NOTCH1 Signalling in Their Genesis
Source: Cancers (Basel). 2021 Apr 27;13(9):2107. doi: 10.3390/cancers13092107 (PMC8123787; doi:10.3390/cancers13092107)
Supplement: Supplementary file 1 [file cancers-13-02107-s001.zip › Cancers-1116687-supplementary.pdf]

cancers

MDPI

Supplementary Materials

Identification of CRYAB⁺ KCNN3⁺ SOX9⁺ Astrocyte-Like and EGFR⁺ PDGFRA⁺ OLIG1⁺ Oligodendrocyte-Like Tumoral Cells in Diffuse IDH1-Mutant Gliomas and Implication of NOTCH1 Signalling in Their Genesis

Meera Augustus, Donovan Pineau, Franck Aimond, Safa Azar, Davide Lecca, Frédérique Scamps, Sophie Muxel, Amélie Darlix, William Ritchie, Catherine Gozé, Valérie Rigau, Hugues Duffau and Jean-Philippe Hugnot

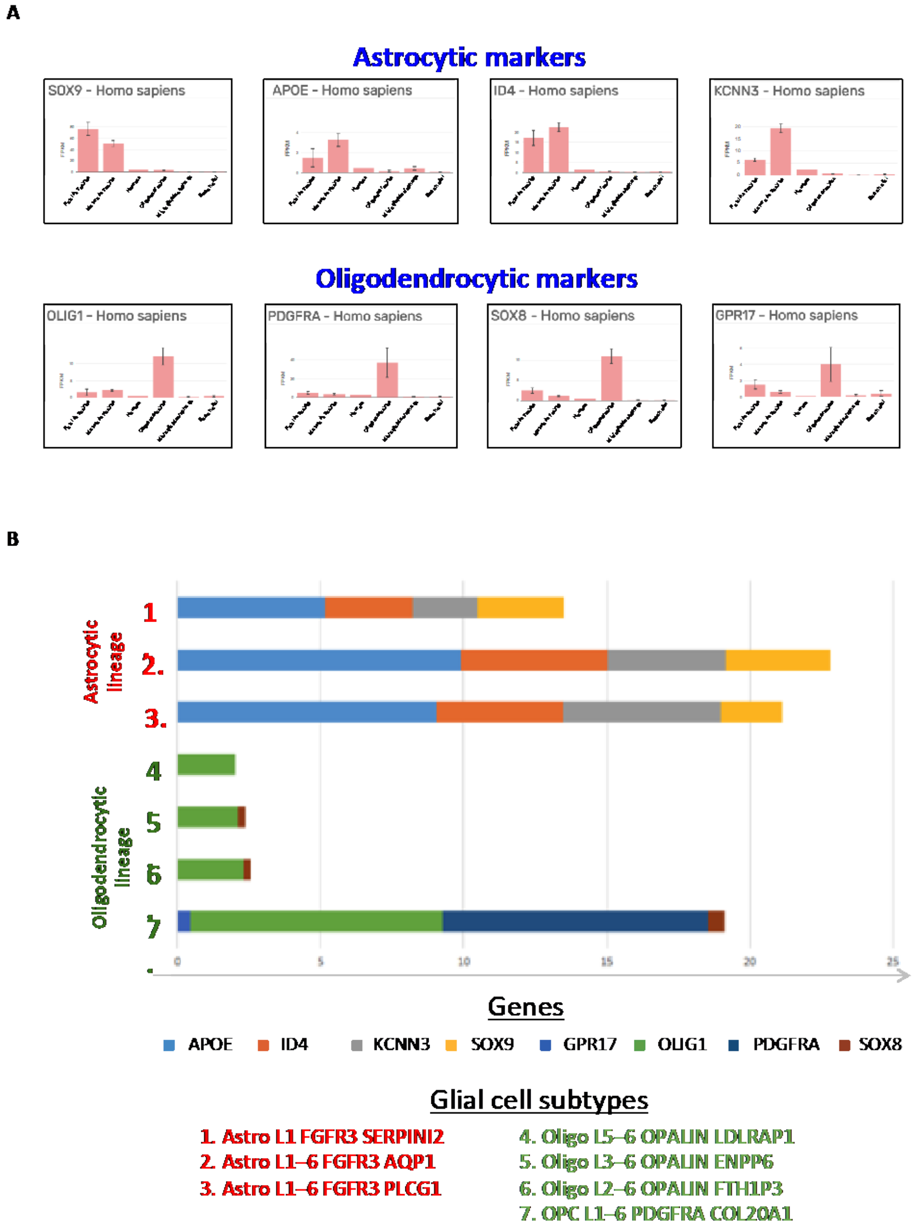

Figure 1. Preferential astrocytic and oligodendrocytic expression of markers selected in the study. (A) RNA levels of indicated genes in different human brain cells. Diagrams were retrieved from the brainrnaseq.org database ([1], Dr. Ben Barres's laboratory). (B) RNA expression of indicated genes

in different glial human brain cells (M1 cortex) measured by single nuclei RNA seq analysis [2]. This bar chart was drawn using data (expression trimmed mean) extracted from the RNA−Seq Human Cell Types Database 2020 (Allen Institute for Brain Science), available at celltypes.brain-map.org/rnaseq/human_m1_10x. RNA level [2]. Horizontal bars show the level of expression of the 9 indicated genes in the 3 human astrocyte− and 4 oligodendrocyte subtypes defined by the single nuclei RNA seq analysis. APOE, ID4, KCNN3 and SOX9 are preferentially expressed in astrocytic cells while GPR17, OLIG1, PDGFRA and SOX8 are mainly expressed in oligodendrocytic cells. .

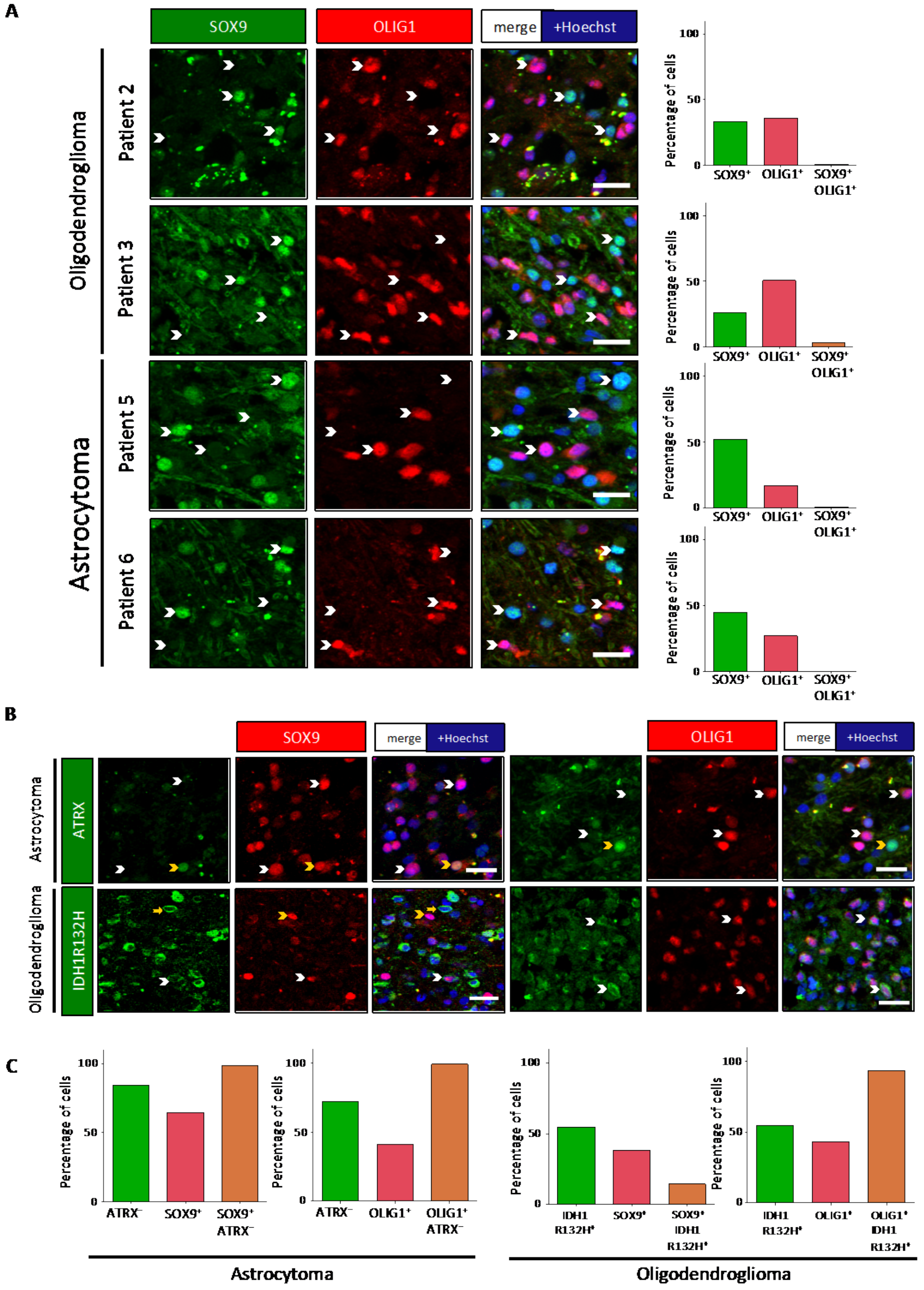

Figure 2. Two non−overlapping cell subpopulations detected in IDH−DGIIG. Double immunofluorescences for indicated markers. (A) Immunofluorescences for SOX9 (green) and OLIG1 (red) on 2 additional oligodendrogliomas and astrocytomas confirmed the presence of two non−overlapping cell subpopulations. White arrowheads mark cells expressing ei-

ther SOX9 or OLIG1 alone. Scale bars=20 µm. Bar diagrams show the percentage of SOX9⁺ cells (green), OLIG1⁺ cells (red) and double positive cells (orange). (B) Tumoral status of SOX9⁺ and OLIG1⁺ cells. Upper images: representative images of an astrocytoma section showing the absence of nuclear expression of ATRX in the majority of the SOX9⁺ and OLIG1⁺ cells. White arrowheads mark ATRX⁻ tumoral cells while yellow arrowheads mark non–tumoral cells expressing nuclear ATRX. Lower images: representative images of an oligodendroglioma section double stained for IDH1 R132H protein and SOX9 or OLIG1. White arrowheads mark OLIG1⁺ and SOX9⁺ cells expressing IDH1 R132H protein. Yellow arrowheads/arrows show single positive cells. The majority of OLIG1⁺ cells express IDH1 R132H protein while this mutated protein is only detected in a minor population of SOX9⁺ cells. Scale bars=20 µm. (C) Quantifications. Left diagrams show the % of ATRX⁻ cells (green), the % of SOX9⁺ or OLIG1⁺ cells (red) and the % of SOX9⁺ or OLIG1⁺ cells that are negative for ATRX (orange) in one astrocytoma. Right diagrams show the % of IDH1 R132H⁺ cells (green), the % of SOX9⁺ or OLIG1⁺ cells (red) and the % of SOX9⁺ or OLIG1⁺ cells in which the IDH1 R132H mutated protein are detected (orange) in one oligodendroglioma.

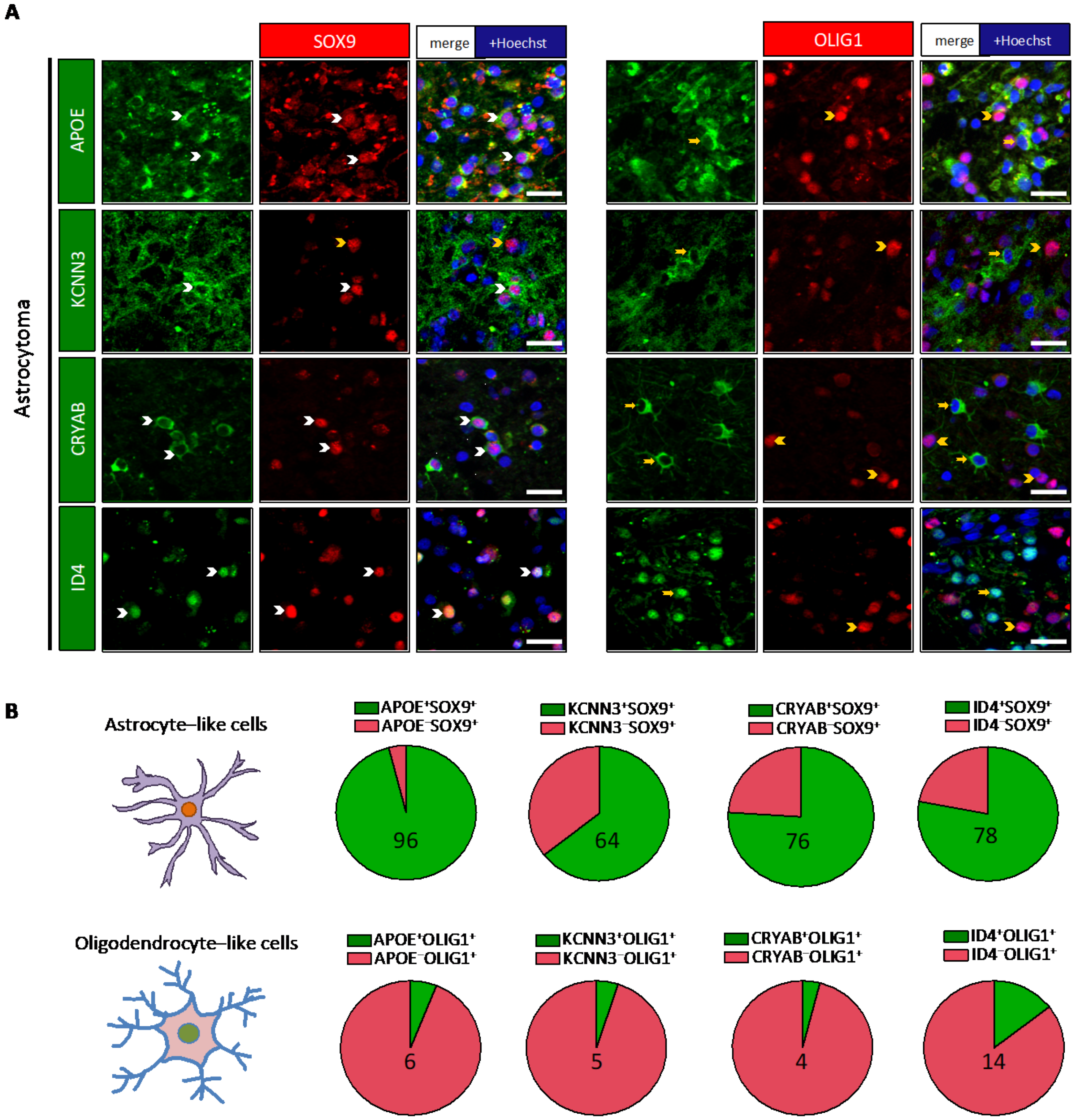

Figure S3. SOX9⁺ cells show specific protein expression. Double immunofluorescences for indicated proteins on one astrocytoma. (A) Stainings for SOX9 (left panel) and OLIG1 (right panel) with APOE, CRYAB, KCNN3 and ID4 showed a preferential expression of these proteins in SOX9⁺ cells. White arrowheads show double positive cells while yellow ar-

rowheads/arrows indicate single positive cells. Scale bars=20 µm. (B) Pie diagrams represent the percentage of double positive (green) and single positive (red) cells in astrocyte–like SOX9⁺ (upper lane) and oligodendrocyte–like OLIG1⁺ (lower lane) cells. Numbers indicate the percentage of double positive cells.

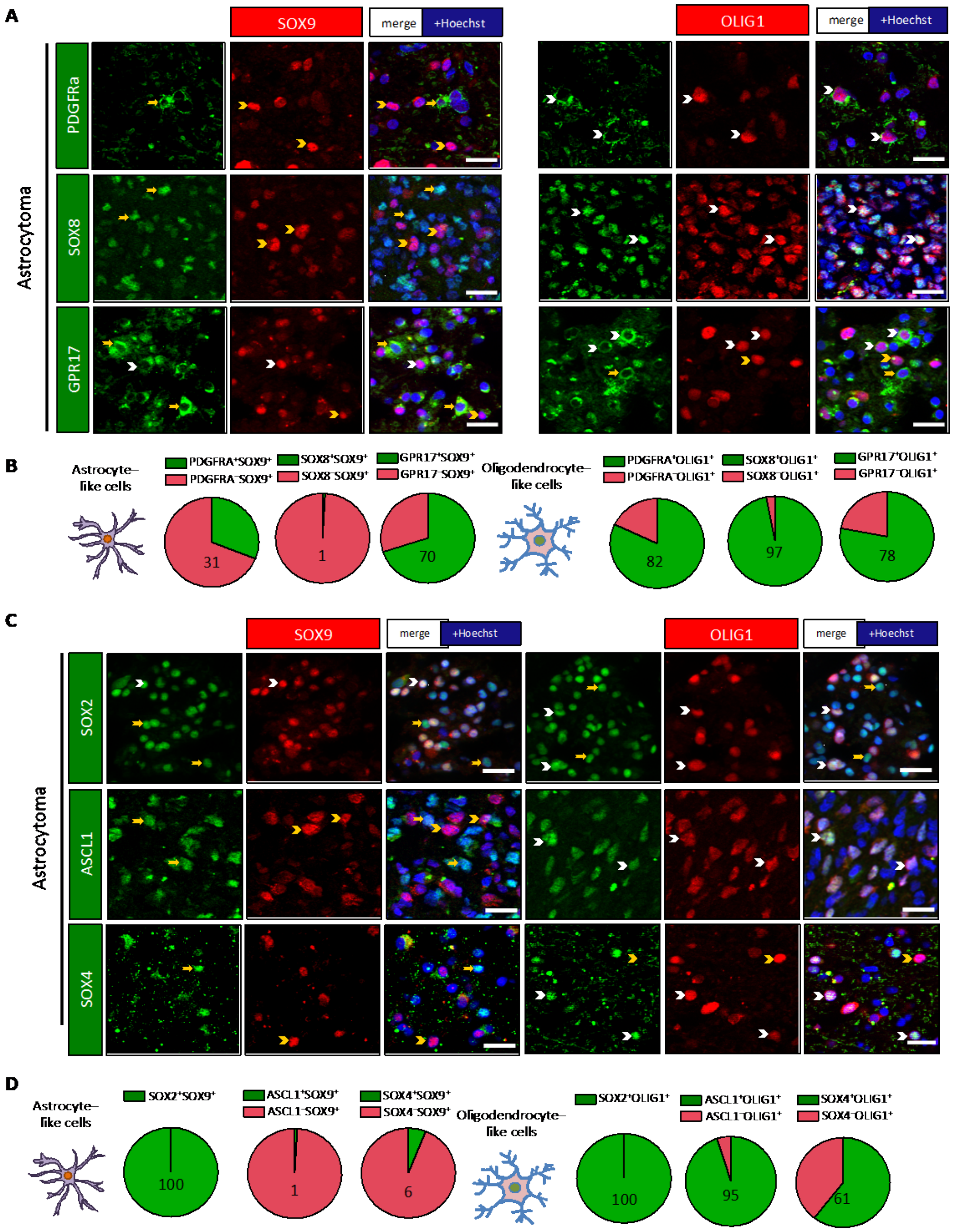

Figure S4. OLIG1⁺ cells express proteins associated to the oligodendrocytic lineage and neural precursor markers. Double immunofluorescences for indicated proteins on one astrocytoma. White arrowheads show double positive cells while yellow arrowheads/arrows show single positive cells. Scale bars=20 µm. (A) Immunofluorescences for PDGFRα and SOX8 revealed their close association with OLIG1⁺ cells while GPR17 was expressed by both SOX9⁺ and OLIG1⁺ cells. (B, D) Pie diagrams represent the percentage of double positive (green) and single positive (red) cells in astrocyte–like SOX9⁺ (left) and oligodendrocyte–like OLIG1⁺ (right) cell populations. Numbers indicate the percentage of double positive cells. (C) Immunofluorescence for ASCL1 and SOX4 show their close association with OLIG1⁺ cells while SOX2 was expressed by both SOX9⁺ and OLIG1⁺ cells.

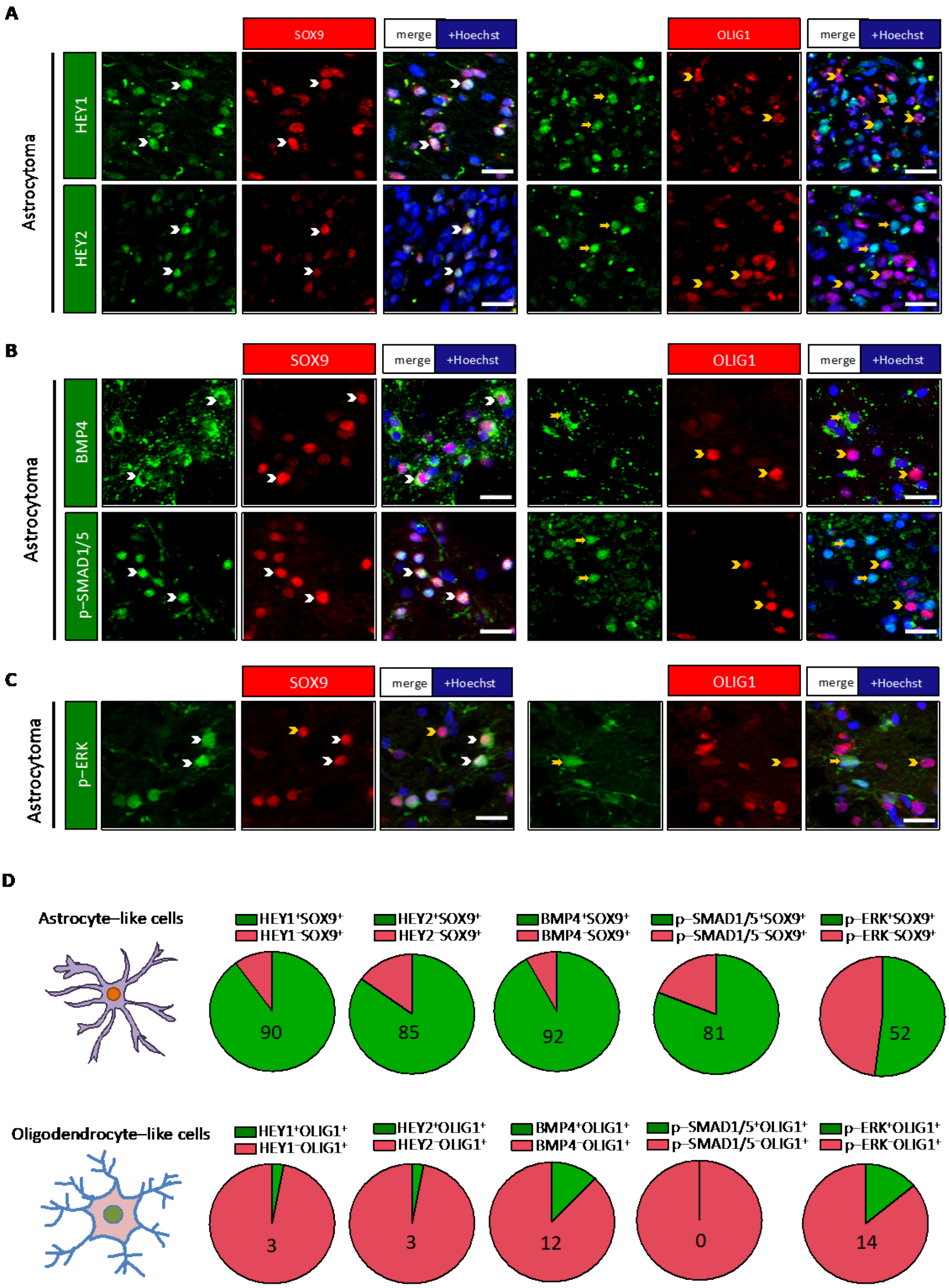

Figure S5. SOX9+ cells specifically express various signalling molecules and receptors. Double immunofluorescences for indicated proteins on one astrocytoma. White arrowheads show double positive cells while yellow arrowheads/arrows show single positive cells. Scale bars=20 μm. (A) Stainings for HEY1 or HEY2 with SOX9 and OLIG1 show their specific expression by SOX9+ cells. (B) Stainings for BMP4 and p–SMAD1/5 revealed their specific expression in SOX9+ cells. (C) Stainings for p–ERK show preferential expression in SOX9+ cells. (D) Pie diagrams represent the percentage of double positive (green) and single positive (red) cells in astrocyte–like SOX9+ (upper lane) and oligodendrocyte–like OLIG1+ (lower lane) cell populations. Numbers indicate the percentage of double positive cells.

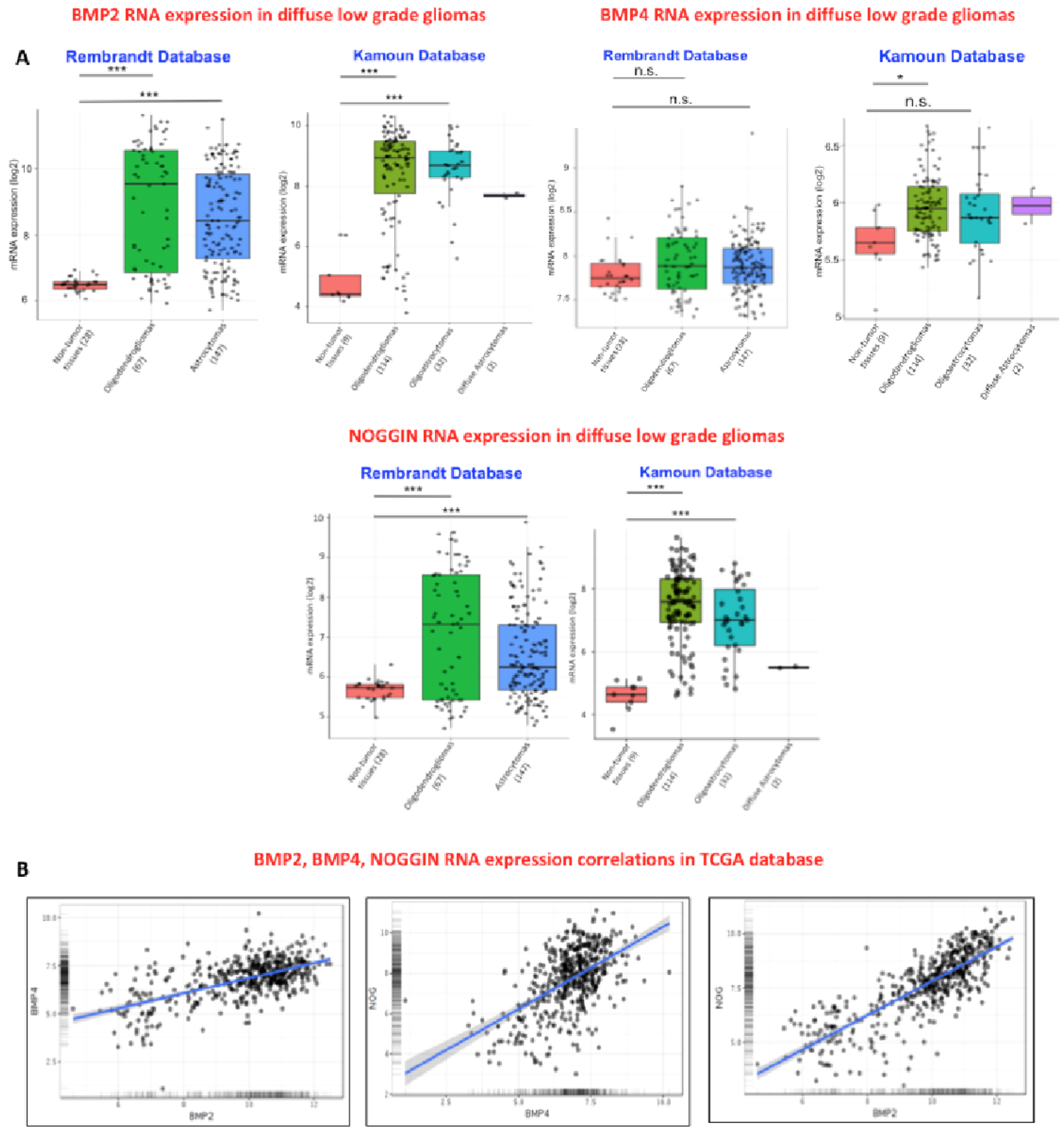

Correlation matrix (LGG-TCGA database)
(Pearson, n=511 cases)

	BMP2	BMP4	NOGGIN
**BMP2**		0.60	0.81
**BMP4**	0.60		0.55
**NOGGIN**	0.81	0.55	

p-values of correlation matrix

	BMP2	BMP4	NOGGIN
**BMP2**		<0.001	<0.001
**BMP4**	<0.001		<0.001
**NOGGIN**	<0.001	<0.001	

Figure S6. Expression profiles for BMP2/4 and NOGGIN mRNA in diffuse low grade glioma databases. (A) RNA levels for BMP2, BMP4 and NOGGIN in non−tumoral brain, oligodendrogliomas, astrocytomas and oligoastrocytomas in Rembrandt [3] and Kamoun [4] databases. Significance was tested using multiple t−tests (p−values were corrected with Bonferroni correction for multiple comparisons). The number of cases is indicated in brackets. (B) Pearson correlation matrix of mRNA expression of BMP2, BMP4 and NOGGIN in the diffuse low grade glioma TCGA database reveals significant correlation. These histograms, scatter dot diagrams and correlation matrix were created with the glioma database mining website GLIOVIS [5].

QPCR analysis of BMP2, BMP4 and NOGGIN expression in diffuse low grade gliomas

A

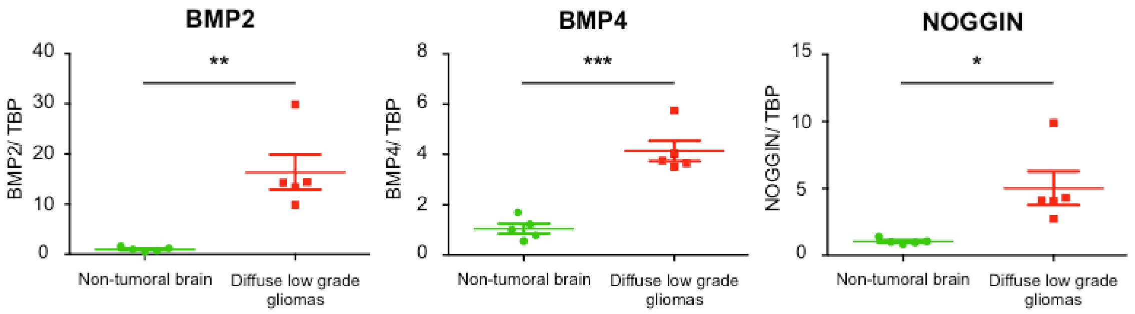

B

Correlation matrix (Pearson, n=5 cases)

	BMP2	BMP4	NOGGIN
**BMP2**		0.91	0.95
**BMP4**	0.91		0.98
**NOGGIN**	0.95	0.98	

p values of correlation matrix

	BMP2	BMP4	NOGGIN
**BMP2**		0.031	0.015
**BMP4**	0.031		0.003
**NOGGIN**	0.015	0.003	

C

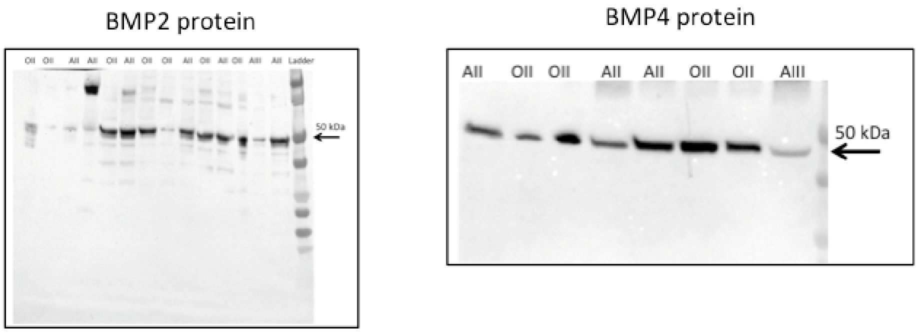

Figure S7. Expression of BMP2/4 and NOGGIN in diffuse low grade gliomas. (A) QPCR data showing increased expression of BMP2, BMP4 and NOGGIN in five diffuse low grade glioma resections (4 grade II oligodendrogliomas and 1 astrocytoma) compared to non–tumoral human brain tissues. Tests are two–tailed t–tests (n=5). (B) Pearson correlation matrix reveals that BMP2, BMP4 and NOGGIN expression are significantly correlated in these diffuse low grade glioma samples. (C) Western blot analysis for BMP2 and BMP4 proteins extracted from IDH1–mutated grade II oligodendrogliomas (OII) and IDH1–mutated grade III or II astrocytomas (AIII, AII). The same amount of proteins (20 μg) per lane was loaded by lane.

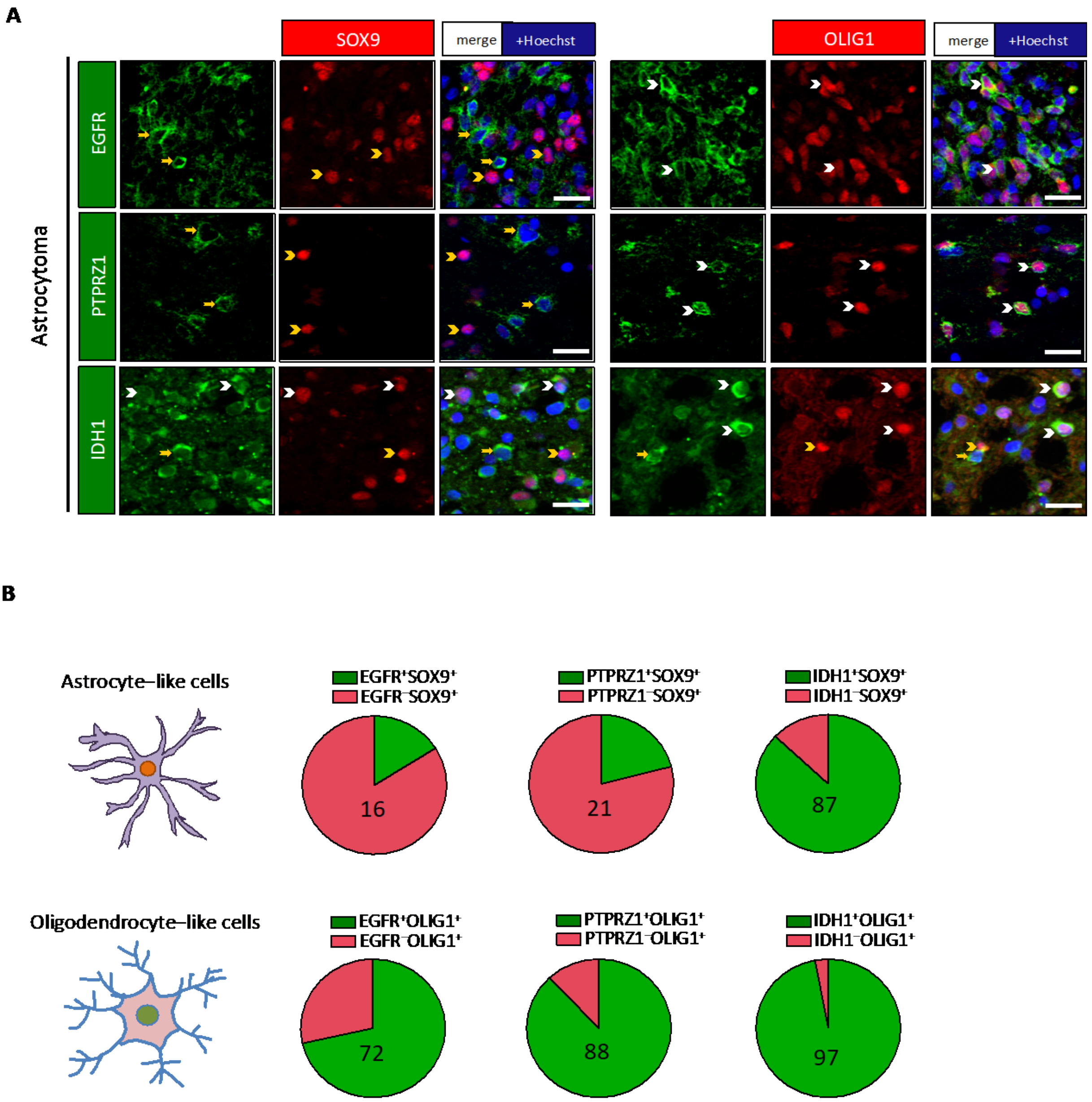

Figure S8. OLIG1⁺ cells specifically express/activate various receptors and IDH1. Double immunofluorescences for indicated proteins on one astrocytoma. White arrowheads show double positive cells while yellow arrowheads/arrows show single positive cells. Scale bars=20 µm. (A) OLIG1⁺ cells preferentially express EGFR and PTPRZ1 compared to SOX9⁺ cells. Expression of IDH1 was closely associated to OLIG1⁺ cells. (B) Pie diagrams represent the percentage of double positive (green) and single positive (red) cells in astrocyte–like SOX9⁺ (upper lane) and oligodendrocyte–like OLIG1⁺ (lower lane) cell populations. Numbers indicate the percentage of double positive cells.

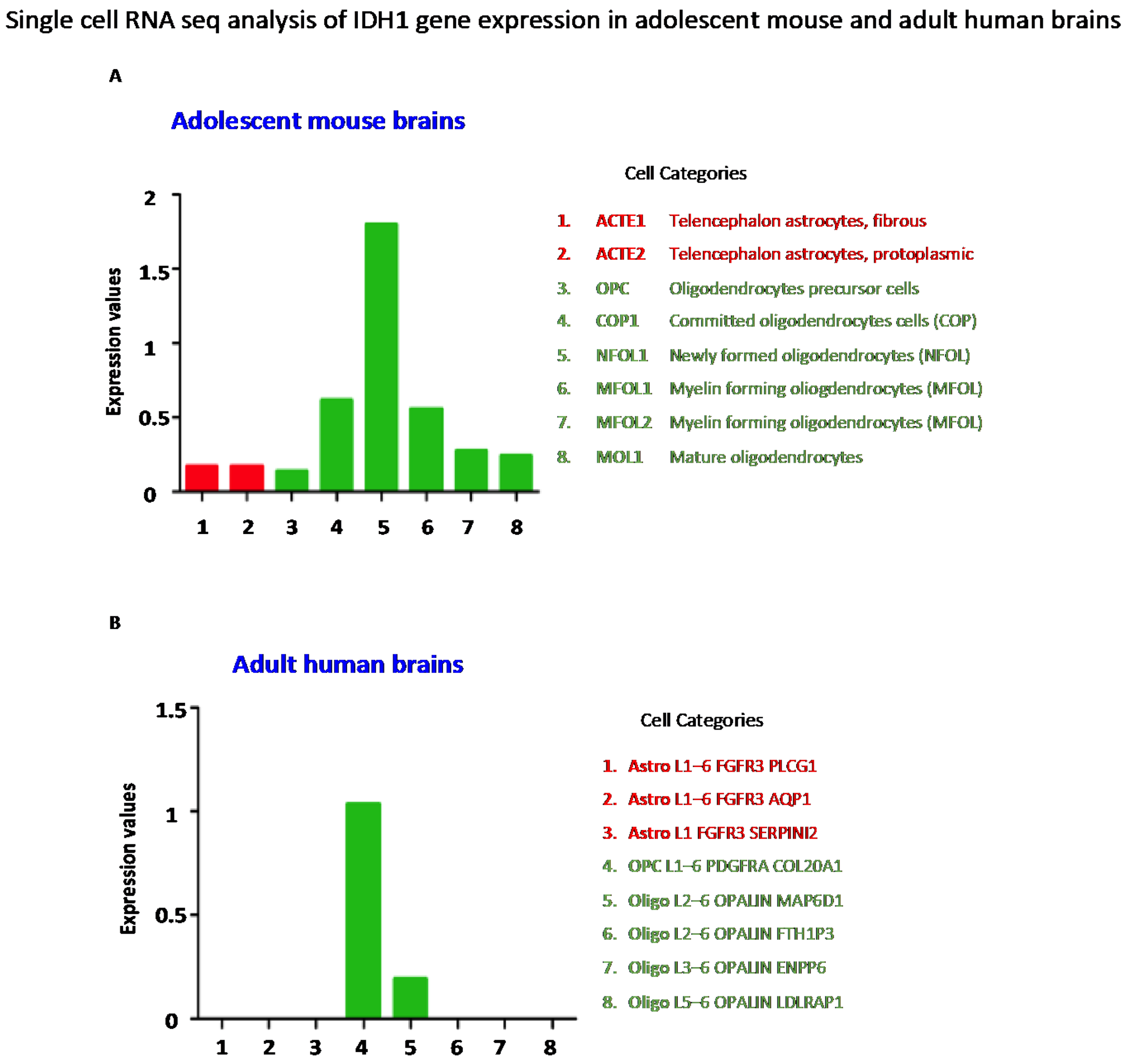

Figure S9. Cell type specific expression of IDH1 in adolescent mouse and adult human brain. Single cell transcriptomic analysis performed in the adolescent mouse and adult human brain reveals oligodendrocytic specific expression of IDH1 gene. (A) In mice, oligodendrocytic cells at different differentiation stages express IDH1 with highest expression in newly formed oligodendrocytes while expression in astrocytes is very low. (B) In the human brain, highest expression is observed in PDGFRA^{+} oligodendrocyte cells (OPC). Mouse and human brain single cell transcriptomics data used to draw these histograms were obtained from the mousebrain.org [6] and Allen brain (celltypes.brain-map.org/rnaseq/human_m1_10x) [2] databases respectively. The different cell categories are those specified in the database websites.

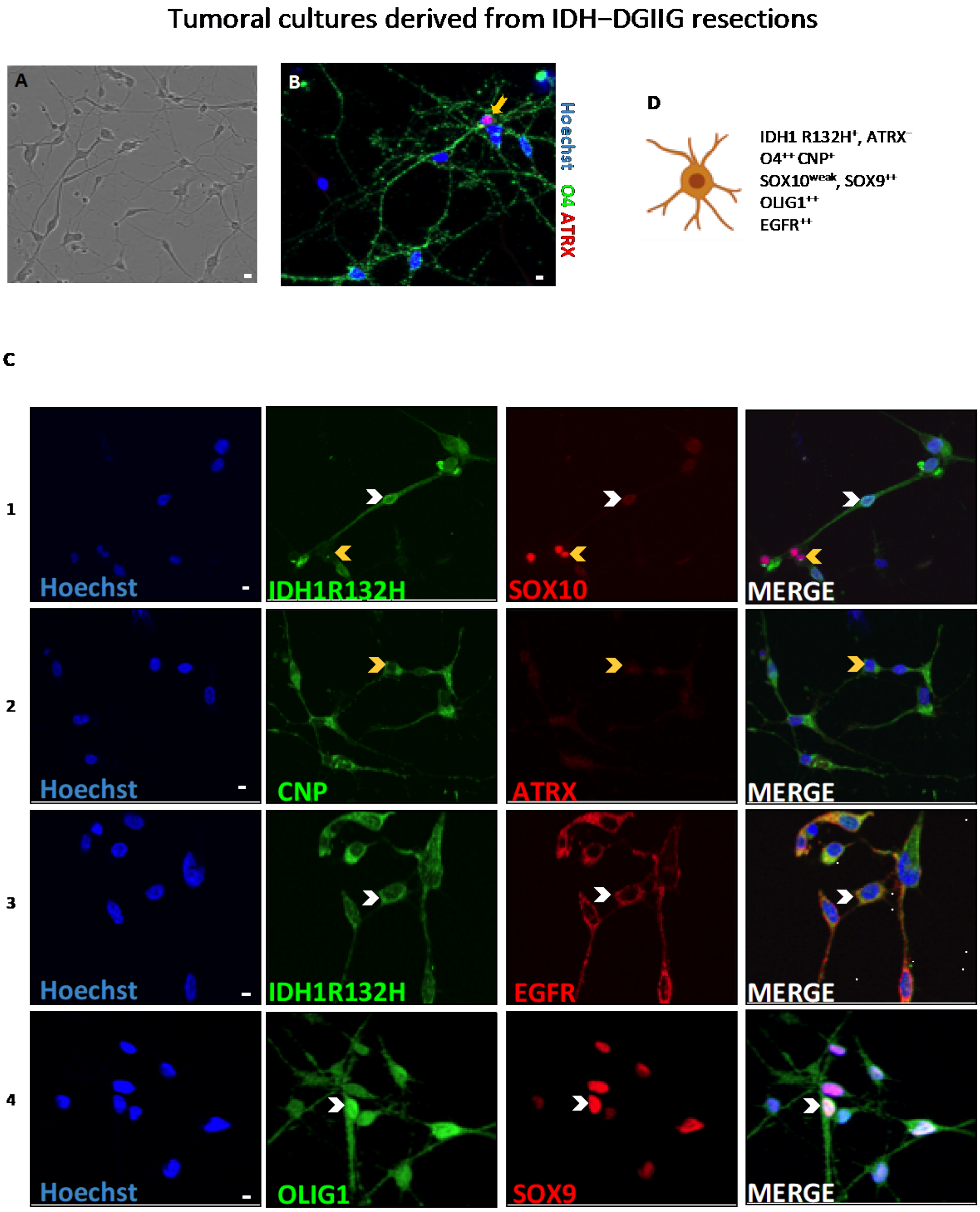

Figure S10. Phenotypic characterization of tumoral O4$^+$ cells isolated from IDH–DGIIG resections. Double immunofluorescence for indicated proteins in a representative culture. (A) Morphology of O4–purified tumoral human cells in culture. Scale bar=10 µm. (B) Double stainings for O4 and ATRX show that the vast majority of cells (>90%) express O4 but not nuclear ATRX and are thus tumoral. One example of a rare O4$^+$ cell maintaining expression of ATRX, and thus probably non–tumoral, is presented to show the quality of staining (yellow arrow). Scale bar=5 µm. (C) Positive staining for IDH1 R132H (lane 1) and absence of nuclear ATRX (lane 2) confirmed the tumoral status of the culture. IDH1 R132H$^+$ cells express SOX10 at low level (white arrowhead, lane 1) in contrast to the rare IDH1 R132H$^-$ non–tumoral cells, which were highly positive for SOX10 (yellow arrowhead, lane 1). Tumoral cells also express EGFR (lane 3), the oligodendrocytic markers CNP (lane 2) and OLIG1 together with SOX9 (white arrowheads) (lane 4). Note that OLIG1 is localized both in the nucleus and cytoplasm. Scale bars=5 µm (D) Graphical summary of the phenotype of tumoral O4–purified cells.

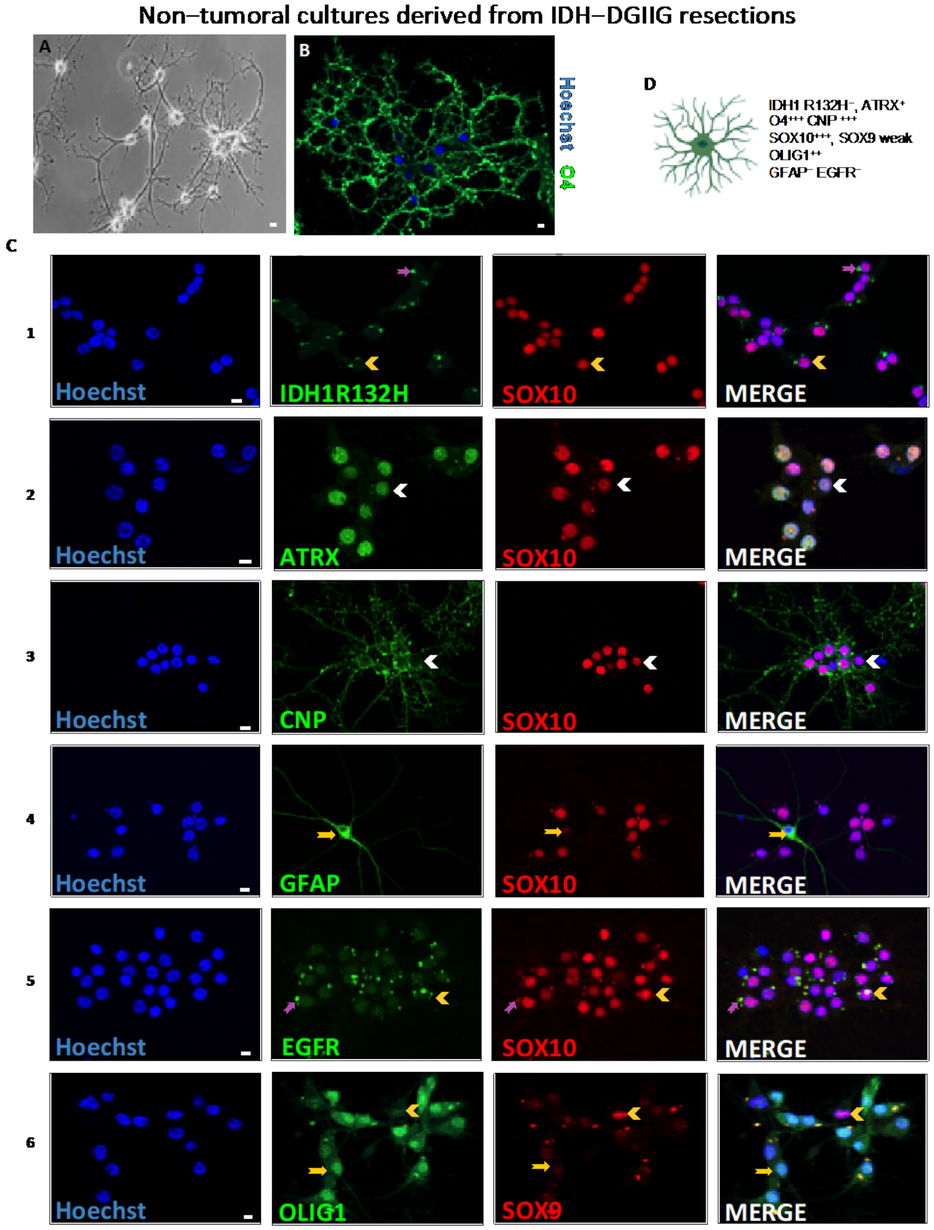

Figure S11. Phenotypic characterization of non–tumoral O4$^+$ cells isolated from IDH–DGIIG samples. (A) Morphology of highly branched non–tumoral cells isolated with O4 purification from a supratotal glioma resection with ATRX and IDH1 R132H mutations. Scale bar=10 µm. (B) Immunofluorescence for O4 shows the very high expression of this antigen. The vast majority of cells (>90%) are O4$^+$. (C) Representative images of immunofluorescences performed on O4–purified cells. White arrowheads represent double positive cells while yellow arrowheads/arrows indicate single positive cells. Of note, non–tumoral cells isolated from IDH–DGIIG resections often–present highly fluorescent green and red auto fluorescent cytoplasmic aggregates (most likely lipofuscin) (pink arrows), which we considered as non–specific signals. Lane 1 and 2: absence of IDH1 R132H protein expression and strong nuclear staining for ATRX show the non–tumoral status of these cells. Lane 3, 4 and 5: these cells are highly positive for oligodendrocytic markers such as CNP, OLIG1, and SOX10 and have small and perfectly round nuclei. Lane 4, 5 and 6: in contrast, the vast majority of these cells (>90%) do not express EGFR, GFAP and SOX9. Two examples of very rare GFAP$^+$ SOX10$^-$ (lane 4) and SOX9$^+$ OLIG1$^-$ (lane 6) cells are presented to show the quality of immunofluorescence (yellow arrow). Scale bars=10 µm (D) Graphical summary of the phenotype of non–tumoral human O4–purified cells.

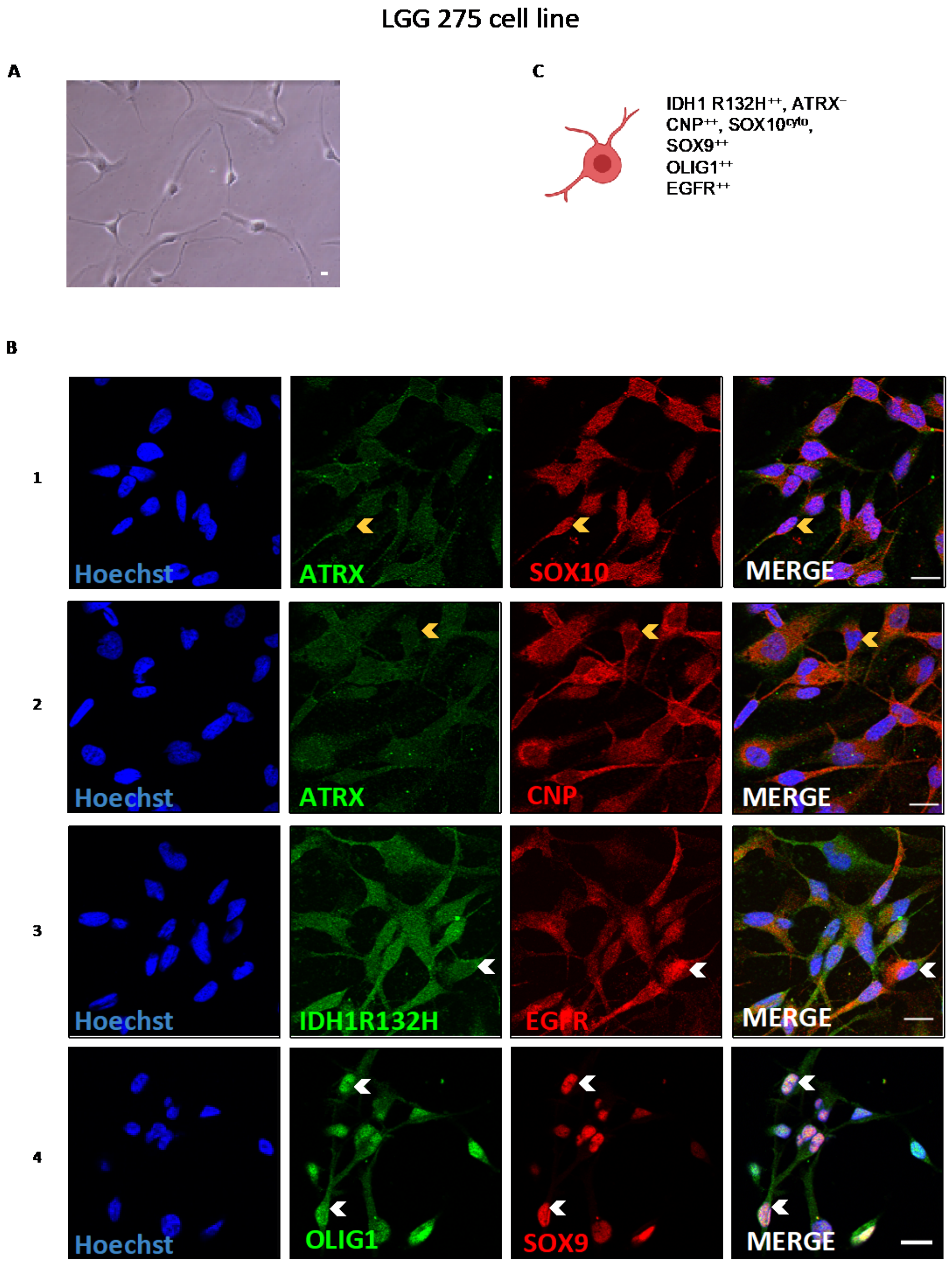

Figure S12. Phenotypic characterization of the LGG275 cell line. (A) Morphology of LGG275 cells. Scale bar=10 µm. (B) Double immunofluorescences for indicated proteins. The tumoral status of these cells is indicated by the detection of IDH1 R132H and absence of nuclear ATRX (lane 1, 2, 3). LGG275 cells show staining for CNP, EGFR, OLIG1 and SOX9 (lane 2, 3, 4). Detection of SOX10 is weak in these cells (lane 1). Note that LGG275 cells show different nuclei size and shape. White arrowheads represent double positive cells while yellow arrowheads indicate single positive cells. Scale bars=20 µm.

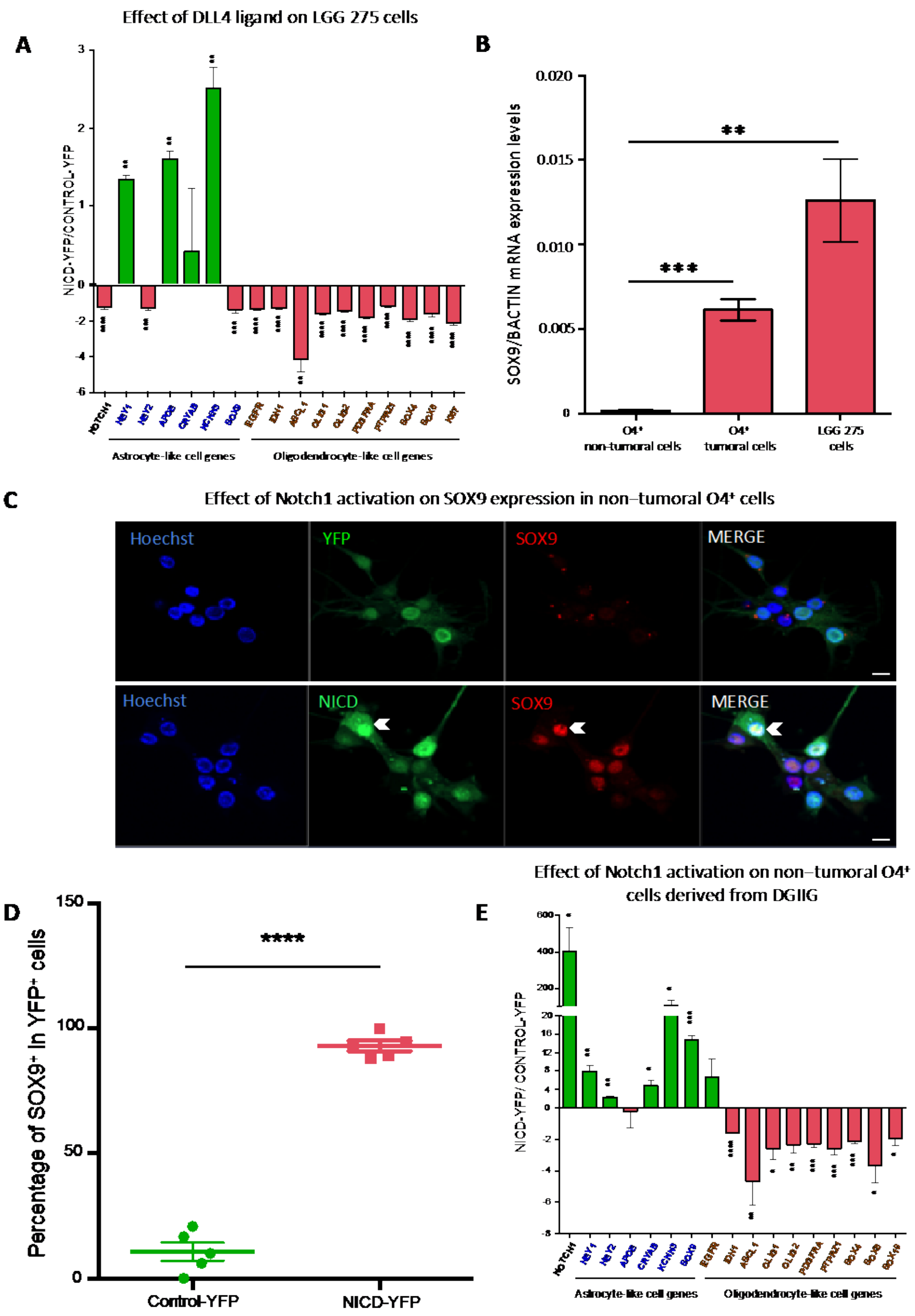

Figure S13. DLL4 effect on cell phenotype and regulation of SOX9 by NOTCH1 signalling. (A) QPCR analysis for 17 genes on LGG275 cells exposed to DLL4 ligand for 5 days. Test=two−tailed t−test, n=3 independent experiments. (B) SOX9 mRNA levels measured by QPCR in O4−purified non−tumoral cells, O4−purified tumoral cells and LGG275 cells. Test=two−tailed t−test, n=3 independent experiments. (C) Immunofluorescence for SOX9 in O4−purified non−tumoral cells transduced with YFP and YFP−NICD lentiviruses. Scale bars=10 µm. (D) Quantification of immunofluorescence presented in (C), n=6 fields, 3 coverslips. Test=two−tailed t−test. (E) QPCR analysis for indicated genes in non−tumoral cells purified with O4 from DGIIG resections (n=3 cases). Values represent the mean +/−S.E.M of gene expression fold change observed in cells transduced with NICD−YFP vs. YFP lentiviruses. Genes in blue and brown are markers found preferentially associated with respectively SOX9+ and OLIG1+ cells on IDH−DGIIG sections. Tests=two−tailed t−tests.

CRYAB, HEY2 and BMP2 co−expression in TCGA_LGG dataset

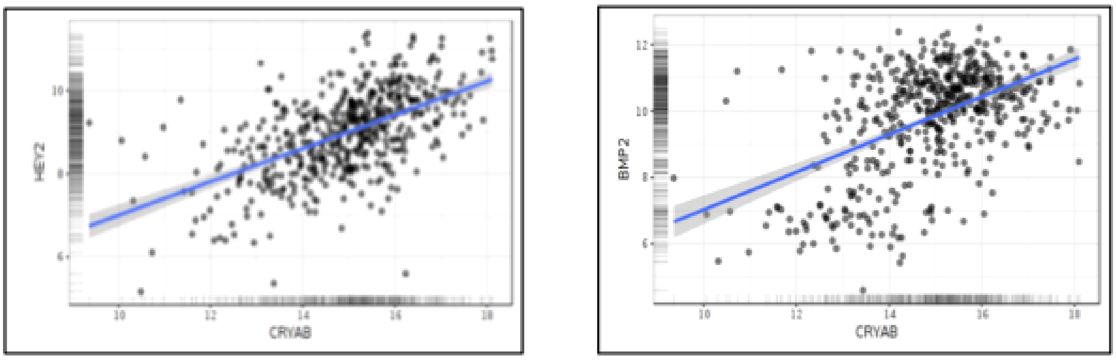

Correlation matrix (Pearson, n=511 cases)

	CRYAB	HEY2	BMP2
**CRYAB**		0.56	0.50
**HEY2**	0.56		0.27
**BMP2**	0.50	0.27	

p values of correlation matrix

	CRYAB	HEY2	BMP2
**CRYAB**		<0.001	<0.001
**HEY2**	<0.001		<0.001
**BMP2**	<0.001	<0.001	

Figure S14. Expression correlation of CRYAB, HEY2 and BMP2 in TCGA database. (A) Upper panels: Expression of CRYAB is highly correlated to HEY2 and BMP2 expression in the diffuse low−grade gliomas TCGA database. Lower panels: Pearson correlation matrix and p−values for CRYAB, HEY2 and BMP2. These scatter dot diagrams and correlation matrix were created with the glioma database mining website GLIOVIS [5].

Table S1. Detailed information of the cases used in the article.

Tumor	Techniques	Figure	Age	Gender	Diagnosis (subtype, grade)	IDH1 mutation	ATRX	1p/19q deletion
Case 1 LGG 236	IF	1, S2B, 2, 3, 4,	49	F	Oligodendroglioma II	IDH1R132H	Preserved	Co−deleted
Case 2 LGG 253	IF	S2A	26	M	Oligodendroglioma II	IDH1R132H	Preserved	Co−deleted
Case 3 LGG 39	IF	S2A	67	M	Oligodendroglioma II	IDH1R132H	Preserved	Co−deleted
Case 4 LGG 234	IF	1, S2B, S3, S4, S5, S8	32	M	Astrocytoma Grade II	IDH1R132H	Loss	No co−deletion
Case 5 LGG 244	IF	S2A	40	M	Astrocytoma II	IDH1R132H	Loss	No co−deletion
Case 6 LGG 188	IF	S2A	42	F	Astrocytoma II	IDH1R132H	Loss	No co−deletion
Case 7 LGG 270	Tumoral cells qPCR−YFP, NICD	5A, S10	38	M	Astrocytoma II	IDH1R132H	Loss	No co−deletion
Case 8 LGG275	Cell line	5B−E, 6A−E, S12, S13A	40	F	Astrocytoma II	IDH1R132H	Loss	No co−deletion
Case 9 LGG 289	Tumoral cells qPCR−YFP, NICD, Cell characterization	5A, S10	47	M	Astrocytoma II	IDH1R132H	Loss	No co−deletion
Case 10 LGG 312	Tumoral cells qPCR−YFP, NICD	5A, S10	31	F	Astrocytoma II	IDH1R132H	Loss	No co−deletion
Case 11 LGG 318	Tumoral cells qPCR−YFP, NICD	5A, S10	26	M	Astrocytoma II	IDH1R132G	Loss	No co−deletion
Case 12 LGG 307	Non−tumoral cells qPCR−YFP, NICD	S11, S13B−E	52	F	Oligodendroglioma II	IDH1wt	No Loss	Co−deleted
Case 13 LGG 335	Non−tumoral cells qPCR−YFP, NICD Cell characterization	S11, S13B−E	30	M	Oligodendroglioma II	IDH1R132H	No Loss	Co−deleted
Case 14 LGG 338	Non−tumoral cells qPCR−YFP, NICD Cell characterization	S11, S13B−E	41	F	Astrocytoma II	IDH1R132H	Loss	No co−deletion

Table S2. List of cellular markers and supporting references for their specific expression.

Protein Name (short form)	Full form	Astrocyte/Oligodendrocyte/progenitor cell	Functions	Supporting references
APOE	Apolipoprotein E	Astrocytes		[7]
CRYAB	Alpha−crystallin B chain			[8,9]
GPR17	G Protein−Coupled Receptor 17	Oligodendrocytes	GPR17 is a downstream target of OLIG2, regulates oligodendrocyte survival and is involved in timing myelination	[10,11]
ID4	Inhibitor of differentiation 4		ID4 is expressed in cells with astrocytic features in oligodendroglioma and astrocytomas	[12,13]
KCNN3/SK3/KCa2.3	Small conductance calcium−activated potassium channel 3		KCNN3 is upregulated in diffuse astrocytoma<br><br>KCNN3 is expressed by GFAP positive astrocytes	[14]<br><br>[15]
MASH1/ ASCL1	Achaete−scute homolog 1			[16−18]
OLIG1	Oligodendrocyte Transcription Factor 1	Oligodendrocytes		[19]
PDGFRA	Platelet−derived growth factor receptor alpha		OPC marker	[20,21]
PTPRZ1	Receptor−type tyrosine−protein phosphatase zeta	Oligodendrocytes		[22]
SOX8	SRY−Box Transcription Factor 8	Oligodendrocytes	Specific marker of oligodendrocytes according to Ben Barres database	[23]<br><br>[1]
SOX9	SRY−Box Transcription Factor 9	Astrocytes		[24]

Table S3. PCR primer pairs used for quantitative RT−PCR.

Gene	Forward primer	Reverse primer
ACTB	GGACTTCGAGCAAGAGATGG	AGCACTGTGTTGGCGTACAG
APOE	GGTCGCTTTTGGGATTACCT	TTCCTCCAGTTCCGATTTGT
BMP2	TGTATCGCAGGCACTCAGGTCA	CCACTCGTTTCTGGTAGTTCTTC
BMP4	CTGGTCTTGAGTATCCTGAGCG	TCACCTCGTTCTCAGGGATGCT
CRYAB	TCATCTCCAGGGAGTTCCAC	AGGACCCCATCAGATGACAG
EGFR	GTGTGCCCACTACATTGACG	CTTCCAGACCAGGGTGTTGT
GPR17	GGAAGAACAACCCCTGAACA	TCCCTCTTCTGGGTCATTTG
HEY1	TGTTTGGTTCAAGGCAGCTC	TGATGCACTGCTGGATGGTA
HEY2	GCACCCTGAAGGTAGCCATA	AGTTACCGAGCTGCCTTGAA
IDH1	AGTCTGCAAGACTGGGAGGA	CAGAACCGCCACTGATTTTT
KCNN3	CTGCCGCCAAAATAAACATT	GCCTGGCACAAGCTTTCTAC
MASH1	CAAGAGAGCGCAGCCTTAGT	CTGGCGCCTTCTTGTTTCTA
MKI67	CCCCCACCAGAACTAACAGA	ACTTTGATGCCCTCATCACC
NOGGIN	TCGAACACCCAGACCCTATC	ATGAAGCCTGGGTCGTAGTG
NOTCH1	TCCACCAGTTTGAATGGTCA	CGCAGAGGGTTGTATTGGTT
OLIG1	CGCAGAGAGTTTTCGCTCTT	GCGGTTGGTTTTCGTTTTTA
OLIG2	GACAAGCTAGGAGGCAGTGG	CGGCTCTGTCATTTGCTTCT
PDGFRA	GCTGATCCGTGCTAAGGAAG	CGACCAAGTCCAGAATGGAT
PTPRZ1	CAATCGCATAGGGACGAAAT	AGTGACTGGTTGGGAAGTGG
SOX10	AGCCCAGGTGAAGACAGAGA	TGTAGGCGATCTGTGAGGTG
SOX4	GCACTAGGACGTCTGCCTTT	ACACGGCATATTGCACAGGA
SOX8	TGATTCACCTGCACTGCTTC	AGCAACTTCTCGGCTGTGTT
SOX9	GGAATGTTTCAGCAGCCAAT	TGGTGTTCTGAGAGGCACAG

Table S4. Antibodies used for immunostaining.

Antibody	Species	References	Suppliers	Dilution
Alpha B Crystallin (CRYAB)	Rabbit	15808−1−AP	Proteintech	1:300
APOE	Mouse	sc−13521	Santa Cruz	1:200
ATRX	Rabbit	HPA−001906	Sigma	1:500
BMP4	Rabbit	NBP1−95882 (EPR6211)	Novus Biological	1:100
CNPase	Mouse	C5922	Sigma	1:100
EGFR	Rabbit	4267	Cell Signaling	1:50
GFAP	Rabbit	Z0334	Dako	1:5000
GPR17	Rabbit	Gift from Davide Lecca		1:100
HEY1	Rabbit	GTX118007	GeneTex	1:200
HEY2	Rabbit	10597−I−AP	Proteintech	1:300
ID4	Rabbit	BCH−9/82−12	Biocheck	1:200
IDH1	Rabbit	8137	Cell Signaling	1:500
IDH1R132H	Mouse	DIA−H09	Dianova	1:100
KCNN3 (SK3)	Rabbit	APC−025	Alomone Labs	1:1000
MASH1/ASCL1	Mouse	556604	BD Biosciences	1:100
MASH1/ASCL1	Rabbit	ab211327	Abcam	1:100
MKI67	Mouse	556003	BD Pharmingen	1:500
NICD	Sheep	AF3647	R&D Systems	1:200
O4	Mouse		supernatant from hybridoma	1:2
OLIG1	Goat	AF2417	R&D Systems	1:300
p−ERK	Rabbit	9910	Cell Signaling	1:200
p−SMAD1/5	Rabbit	9516	Cell Signaling	1:800
PDGFR◎	Rabbit	3174	Cell Signaling	1:200
PTPR◎ ◎	Rabbit	sc−25432	Santa Cruz	1:200
SOX10	Mouse	MAB2864	R&D Systems	1:200
SOX10	Rabbit	ab155279	Abcam	1:200
SOX2	Rabbit	23064	Cell Signaling	1:200
SOX4	Mouse	AMab91378	Atlas	1:250
SOX8	Rabbit	20627−1−AP	Proteintech	1:300
SOX9	Rabbit	82630S	Cell Signaling	1:300
SOX9	Goat	AF3075−SP	R&D Systems	1:300

Table S5. List of Abbreviations used in the article.

APOE	Apolipoprotein E
ASCL1/MASH1	Achaete−Scute Family BHLH Transcription Factor 1
ATRX	Alpha−Thalassemia/ Mental Retardation Syndrom X−linked
BMP	Bone Morphogenetic protein
CIC	Capicua Transcriptional Repressor
CNP	2',3'−Cyclic−nucleotide 3'−phosphodiesterase
CRYAB	Crystallin Alpha B
DLL4	Delta−like 4
EGFR	Epithelial Growth Factor Receptor
ETNPPL	Ethanolamine−Phosphate Phospho−Lyase
FUBP1	Far−upstream element (FUSE) binding protein 1
GFAP	Glial fibrillary acidic protein
GPR17	G−protein coupled receptor 17
HEY	Hairy/enhancer−of−split related with YRPW motif protein
ID4	Inhibitors of Differentiation 4
IDH1	Isocitrate Dehydrogenase I
IDH−DGIIG	Diffuse grade II IDH−mutant glioma
KCNN3/ SK3	Potassium Calcium−Activated Channel Subfamily N Member 3/ small conductance calcium−activated potassium channel 3
MAPK	Mitogen−activated protein kinase
NICD	Notch intracellular domain
NOG	Noggin
O4	Oligodendrocyte Marker 4
OLIG1	Oligodendrocyte Transcription Factor 1
PDGFRA	Platelet−derived Growth factor Receptor alpha
PI3K−AKT−mTOR	Phosphoinositide 3−kinases− Akt− mechanistc target of rapamycin
PTN	Pleiotrophin
PTPRZ1	Receptor−type tyrosine−protein phosphatase zeta 1
STAT3	Signal transducer and activator of trasncription 3
TERT	Telomerase reverse transcriptase

References for Supplementary Information

1. Zhang, Y.; Sloan, S.A.; Clarke, L.E.; Caneda, C.; Plaza, C.A.; Blumenthal, P.D.; Vogel, H.; Steinberg, G.K.; Edwards, M.S.; Li, G.; et al. Purification and Characterization of Progenitor and Mature Human Astrocytes Reveals Transcriptional and Functional Differences with Mouse. *Neuron* **2016**, *89*, 37–53, doi:10.1016/j.neuron.2015.11.013.
2. Hodge, R.D.; Bakken, T.E.; Miller, J.A.; Smith, K.A.; Barkan, E.R.; Graybuck, L.T.; Close, J.L.; Long, B.; Johansen, N.; Penn, O.; et al. Conserved cell types with divergent features in human versus mouse cortex. *Nat. Cell Biol.* **2019**, *573*, 61–68, doi:10.1038/s41586-019-1506-7.
3. Madhavan, S.; Zenklusen, J.-C.; Kotliarov, Y.; Sahni, H.; Fine, H.A.; Buetow, K. Rembrandt: Helping Personalized Medicine Become a Reality through Integrative Translational Research. *Mol. Cancer Res.* **2009**, *7*, 157–167, doi:10.1158/1541-7786.mcr-08-0435.
4. Kamoun, A.; Network, P.; Idbaih, A.; Dehais, C.; Elarouci, N.; Carpentier, C.; Letouzé, E.; Colin, C.; Mokhtari, K.; Jouvet, A.; et al. Integrated multi-omics analysis of oligodendroglial tumours identifies three subgroups of 1p/19q co-deleted gliomas. *Nat. Commun.* **2016**, *7*, 11263, doi:10.1038/ncomms11263.
5. Bowman, R.L.; Wang, Q.; Carro, A.; Verhaak, R.G.; Squatrito, M. GlioVis data portal for visualization and analysis of brain tumor expression datasets. *Neuro. Oncol.* **2017**, *19*, 139–141.
6. Zeisel, A.; Hochgerner, H.; Lönnerberg, P.; Johnsson, A.; Memic, F.; van der Zwan, J.; Häring, M.; Braun, E.; Borm, L.E.; La Manno, G.; et al. Molecular Architecture of the Mouse Nervous System. *Cell* **2018**, *174*, 999–1014.e22, doi:10.1016/j.cell.2018.06.021.
7. Pitas, R.E.; Boyles, J.K.; Lee, S.H.; Foss, D.; Mahley, R.W. Astrocytes synthesize apolipoprotein E and metabolize apolipoprotein E-containing lipoproteins. *Biochim. et Biophys. Acta (BBA) - Lipids Lipid Metab.* **1987**, *917*, 148–161, doi:10.1016/0005-2760(87)90295-5.
8. Avliyakulov, N.K.; Rajavel, K.S.; Le, K.M.T.; Guo, L.; Mirsadraei, L.; Yong, W.H.; Liau, L.M.; Li, S.; Lai, A.; Nghiemphu, P.L.; et al. C-terminally truncated form of αB-crystallin is associated with IDH1 R132H mutation in anaplastic astrocytoma. *J. Neuro-Oncology* **2014**, *117*, 53–65, doi:10.1007/s11060-014-1371-z.
9. Goplen, D.; Bougnaud, S.; Rajcevic, U.; Bøe, S.O.; Skaftnesmo, K.O.; Voges, J.; Enger, P. Ø.; Wang, J.; Tysnes, B.B.; Laerum, O.D.; et al. αB-Crystallin Is Elevated in Highly Infiltrative Apoptosis-Resistant Glioblastoma Cells. *Am. J. Pathol.* **2010**, *177*, 1618–1628, doi:10.2353/ajpath.2010.090063.
10. Chen, Y.; Wu, H.; Wang, S.; Koito, H.; Li, J.; Ye, F.; Hoang, J.; Escobar, S.S.; Gow, A.; Arnett, H.A.; et al. The oligodendrocyte-specific G protein–coupled receptor GPR17 is a cell-intrinsic timer of myelination. *Nat. Neurosci.* **2009**, *12*, 1398–1406, doi:10.1038/nn.2410.
11. Ou, Z.; Sun, Y.; Lin, L.; You, N.; Liu, X.; Li, H.; Ma, Y.; Cao, L.; Han, Y.; Liu, M.; et al. Olig2-Targeted G-Protein-Coupled Receptor Gpr17 Regulates Oligodendrocyte Survival in Response to Lysolecithin-Induced Demyelination. *J. Neurosci.* **2016**, *36*, 10560–10573, doi:10.1523/JNEUROSCI.0898-16.2016.
12. Liang, Y.; Bollen, A.W.; Nicholas, M.K.; Gupta, N. Id4 and FABP7 are preferentially expressed in cells with astrocytic features in oligodendrogliomas and oligoastrocytomas. *BMC Clin. Pathol.* **2005**, *5*, 6, doi:10.1186/1472-6890-5-6.
13. Samanta, J. Interactions between ID and OLIG proteins mediate the inhibitory effects of BMP4 on oligodendroglial differentiation. *Dev.* **2004**, *131*, 4131–4142, doi:10.1242/dev.01273.
14. Rorive, S.; Maris, C.; Debeir, O.; Sandras, M.F.; Vidaud, M.; Bièche, I.; Salmon, I.; Decaestecker, C. Exploring the Distinctive Biological Characteristics of Pilocytic and Low-Grade Diffuse Astrocytomas Using Microarray Gene Expression Profiles. *J. Neuropathol. Exp. Neurol.* **2006**, *65*, 794–807, doi:10.1097/01.jnen.0000228203.12292.a1.
15. Armstrong, W.E.; Rubrum, A.; Teruyama, R.; Bond, C.T.; Adelman, J.P. Immunocytochemical localization of small-conductance, calcium-dependent potassium channels in astrocytes of the rat supraoptic nucleus. *J. Comp. Neurol.* **2005**, *491*, 175–185, doi:10.1002/cne.20679.
16. Nakatani, H.; Martin, E.; Hassani, H.; Clavairoly, A.; Maire, C.L.; Viadieu, A.; Kerninon, C.; Delmasure, A.; Frah, M.; Weber, M.; et al. Ascl1/Mash1 Promotes Brain Oligodendrogenesis during Myelination and Remyelination. *J. Neurosci.* **2013**, *33*, 9752–9768, doi:10.1523/jneurosci.0805-13.2013.
17. Rousseau, A.; Nutt, C.L.; Betensky, R.A.; Iafrate, A.J.; Han, B.M.; Ligon, K.L.; Rowitch, D.H.; Louis, D.N. Expression of Oligodendroglial and Astrocytic Lineage Markers in Diffuse Gliomas. *J. Neuropathol. Exp. Neurol.* **2006**, *65*, 1149–1156, doi:10.1097/01.jnen.0000248543.90304.2b.
18. Sugimori, M.; Nagao, M.; Parras, C.M.; Nakatani, H.; Lebel, M.; Guillemot, F.; Nakafuku, M. Ascl1 is required for oligodendrocyte development in the spinal cord. *Dev.* **2008**, *135*, 1271–1281, doi:10.1242/dev.015370.
19. Othman, A.; Frim, D.M.; Polak, P.; Vujicic, S.; Arnason, B.G.W.; Boullerne, A.I. Olig1 is expressed in human oligodendrocytes during maturation and regeneration. *Glia* **2011**, *59*, 914–926, doi:10.1002/glia.21163.
20. Ellison, J.A.; De Vellis, J. Platelet-derived growth factor receptor is expressed by cells in the early oligodendrocyte lineage. *J. Neurosci. Res.* **1994**, *37*, 116–128, doi:10.1002/jnr.490370116.
21. Zhu, Q.; Zhao, X.; Zheng, K.; Li, H.; Huang, H.; Zhang, Z.; Mastracci, T.; Wegner, M.; Chen, Y.; Sussel, L.; et al. Genetic evidence that Nkx2.2 and Pdgfra are major determinants of the timing of oligodendrocyte differentiation in the developing CNS. *Dev.* **2014**, *141*, 548–555, doi:10.1242/dev.095323.

22. Lamprianou, S.; Chatzopoulou, E.; Thomas, J.-L.; Bouyain, S.; Harroch, S. A complex between contactin-1 and the protein tyrosine phosphatase PTPRZ controls the development of oligodendrocyte precursor cells. *Proc. Natl. Acad. Sci. USA* **2011**, *108*, 17498–17503, doi:10.1073/pnas.1108774108.
23. Azar, S.; Leventoux, N.; Ripoll, C.; Rigau, V.; Gozé, C.; Lorcy, F.; Bauchet, L.; Duffau, H.; Guichet, P.O.; Rothhut, B.; et al. Cellular and molecular characterization of IDH1-mutated diffuse low grade gliomas reveals tumor heterogeneity and absence of EGFR/PDGFRα activation. *Glia* **2017**, *66*, 239–255, doi:10.1002/glia.23240.
24. Sun, W.; Cornwell, A.; Li, J.; Peng, S.; Osorio, M.J.; Aalling, N.; Wang, S.; Benraiss, A.; Lou, N.; Goldman, S.A.; et al. SOX9 Is an Astrocyte-Specific Nuclear Marker in the Adult Brain Outside the Neurogenic Regions. *J. Neurosci.* **2017**, *37*, 4493–4507, doi:10.1523/jneurosci.3199-16.2017.
